# Use of the Syrian Hamster as a New Model of Ebola Virus Disease and Other Viral Hemorrhagic Fevers

**DOI:** 10.3390/v4123754

**Published:** 2012-12-14

**Authors:** Victoria Wahl-Jensen, Laura Bollinger, David Safronetz, Fabian de Kok-Mercado, Dana P. Scott, Hideki Ebihara

**Affiliations:** 1 Integrated Research Facility at Fort Detrick, National Institute of Allergy and Infectious Diseases (NIAID), National Institutes of Health (NIH), National Interagency Biodefense Campus, B-8200 Research Plaza, Fort Detrick, Frederick, Maryland 21702, USA; E-Mail: bollingerl@niaid.nih.gov; 2 Integrated Research Facility at Rocky Mountain Labs, National Institute of Allergy and Infectious Diseases (NIAID), National Institutes of Health (NIH), 903 South 4th Street, Hamilton, Montana 59840, USA; E-Mails: safronetzd@niaid.nih.gov (D.S.); dekokmercadof@niaid.nih.gov (F.d.K.-M.); dana.scott@nih.gov (D.P.S.); ebiharah@niaid.nih.gov (H.E.)

**Keywords:** Ebola, filovirus, hamster model, rodent model, pathogenesis

## Abstract

Historically, mice and guinea pigs have been the rodent models of choice for therapeutic and prophylactic countermeasure testing against Ebola virus disease (EVD). Recently, hamsters have emerged as a novel animal model for the *in vivo* study of EVD. In this review, we discuss the history of the hamster as a research laboratory animal, as well as current benefits and challenges of this model. Availability of immunological reagents is addressed. Salient features of EVD in hamsters, including relevant pathology and coagulation parameters, are compared directly with the mouse, guinea pig and nonhuman primate models.

## 1. Introduction

Ebola virus (EBOV), a member of the family *Filoviridae*, is the etiologic agent of Ebola virus disease (EVD), a severe hemorrhagic fever syndrome with unusually high case-fatality rates, ranging between 65–90%. Filoviruses are emerging/reemerging zoonotic agents that are highly virulent in primates, and the frequency of outbreaks in Africa and Asia and impact on ape populations have been increasing in recent years. Introduction of filoviruses into human populations leads to serious, albeit limited, epidemics. Interhuman transmission occurs by direct person-to-person contact and possibly by fomites and droplets. Filoviruses infect, among others, monocytes, macrophages, dendritic cells, hepatocytes, and endothelial cells. In the infected primate, these viral infections lead to severe cytokine imbalances that impair the innate and adaptive immune responses, disseminated intravascular coagulation (e.g., hemorrhages, thrombi), and organ necroses that result in multi-organ failure and shock. No approved vaccines or effective therapeutics are currently available. Because of the high case-fatality rates of EVD and the lack of an approved vaccine or therapy, EBOV is classified as a category A pathogen requiring biosafety level-4 (BSL-4) biocontainment. 

## 2. Ebola Virus Disease (Humans): Clinical Presentation and Pathogenesis

Ebolaviruses likely enter the body via direct contact (skin abrasions, mucous membranes) or contact with bodily fluids to directly access the vascular system or indirectly access the lymphatic system [1]. Limited human data indicate that monocytes/macrophages and dendritic cells are primary sites of virus replication [2]. Ebolaviruses spread from initial infection sites via macrophages and dendritic cells trafficking to regional lymph nodes, liver, and spleen [1]. After an incubation period of 4–16 days, patients initially present with influenza-like symptoms such as abdominal pain, anorexia, arthralgia, asthenia, back pain, diarrhea, fever, headaches, enlarged lymph nodes, myalgia, nausea, or vomiting [3,4,5]. After approximately 5–7 days, a maculopapular rash usually develops on the face, buttocks, trunk, or arms and later generalizes over almost the entire body. As EVD progresses, more severe and multisystem symptoms include respiratory (e.g., chest pain, terminal tachypnea, cough), vascular (e.g., conjunctival injection, postural hypotension, disseminated intravascular coagulation), urinary (e.g., anuria), and neurologic (e.g., headache, confusion, coma) manifestations [5,6]. In fatal EVD cases, hemorrhagic manifestations are usually striking with hematemesis, hemoptysis, melena, hematuria, epistaxis, and/or widespread petechiae and ecchymoses involving skin, mucous membranes, and internal organs [7,8]. Hemorrhagic manifestations occur approximately 3–4 days postonset of influenza-like symptoms and progress, in fatal cases, to disseminated intravascular coagulation (decrease of clotting factors, thrombocytopenia, increased fibrin degradation products, prolonged thrombin and activated partial thromboplastin times) [5,9,10]. 

Death occurs 3–21 days after disease onset from shock after multi-organ failure (liver, spleen, kidney). Liver damage is characterized by hepatocellular necrosis, sinusoidal dilation and congestion (hepatomegaly), and elevated concentrations of aspartate transaminase (AST), alanine aminotransferase (ALT), alkaline phosphatase (ALP), and γ-glutamyl transferase (GGT) [2,3,4,10]. Within the spleen, marked hyperemia and splenomegaly, cellular depletion of the red pulp, and/or marked atrophy of the lymphoid follicles are observed [8,11]. Lymphoid hypoplasia or depletion has been noted in patients with EVD [2,8,11]. Leukopenia (lymphopenia) and granulocytosis are present initially in patients with EVD, and as the disease progresses, leukocytosis results from an increase in immature granulocytes and atypical lymphocytes [4,12,13]. Acute renal tubular necrosis and calcification of renal tubules and glomerular tufts are noted [2,3,14]. Creatinine and urea concentrations increase prior to renal failure, and hypokalemia is typical due to diarrhea and/or vomiting. Lung hemorrhage (hemoptysis) progresses to focal atelectasis and is accompanied by interstitial pneumonitis [2,11].

Infected monocytes/macrophages release soluble mediators including proinflammatory cytokines and vasoactive substances [15]. These cytokines recruit additional macrophages to infected areas and could increase the number of target cells available for virus infection, further amplifying an already dysregulated host response [16]. EBOV-infected patients with a fatal outcome exhibited increased concentrations of interferon (IFN)-γ, IFN-α, interleukin (IL)-2, IL-6, IL-8, IL-10, IL-1 receptor antagonist, macrophage inflammatory protein-1β, neopterin, or tumor necrosis factor (TNF)-α, although differences in cytokine release were apparent between variants of EBOV [15,17,18]. 

## 3. Existing Animal Models of EVD

The development of animal models that accurately reflect human disease is critical to our understanding of the pathogenesis of EVD and evaluation of countermeasures against filoviruses. Because of the sporadic and geographically isolated nature of EVD outbreaks, clinical efficacy studies may not be feasible. Clinical data and access to human tissues from fatal cases are limited. 

Another option for licensing new drugs and vaccines for EVD is extrapolation of data derived from accurate, validated animal models supported by human safety evaluation data and pharmacokinetic information. The “Animal Rule” from the U.S. Food and Drug Administration [19] requires that a countermeasure be evaluated in animal models in which the route and dose of virus administration, time to onset of disease, and time course/progression of disease optimally mimic the pathophysiology of human disease. One of the challenges with this regulatory pathway is the development of animal models that recapitulate human disease, as data on the clinical presentation of EVD disease in humans are quite limited. 

The nonhuman primate (NHP) model of EVD is the gold-standard for the study of EVD pathogenesis that most closely resembles what we currently know regarding human disease. Guinea pigs and mice are regarded as models for preliminary evaluation of therapeutic interventions against EVD. As wild-type EBOV does not produce appreciable disease in these rodent models, EBOV was adapted by serial passage to produce fatal infection following intraperitoneal (IP) inoculation [20,21]. The pathogenesis of EVD from adapted rodent viruses differs in a number of aspects from EVD in humans and NHPs. Important clinical signs of EVD in humans and NHPs such as fever and maculopapular rash are not present in mice infected with mouse-adapted Ebola virus (MA-EBOV) [20,22]. Fever is present in guinea pigs infected with guinea pig-adapted Ebola virus (GPA-EBOV), but maculopapular rash does not develop in these animals [21]. Mice infected with MA-EBOV do not consistently display coagulation abnormalities (Table 1) [20,23,24]. Compared to mice, guinea pigs infected with GPA-EBOV develop coagulation defects, including a drop in platelet counts and an increase in coagulation time, but fibrin deposition and coagulopathy (*i.e*., disseminated intravascular coagulation) are not as marked as that observed in NHPs [21,25]. 

**Table 1:** Coagulation parameters in animal models of Ebola virus disease ^a^.

Coagulation Parameter	Rhesus Macaque ^b^	Syrian Hamster ^c^	Guinea Pig ^d^	Mouse ^c^
**Increased prothrombin time (PT)**	(++) [26,27]	(+++)	(+++)	(-) [20,27]
**Increased activated partial thromboplastin time (aPTT)**	(++) [26,27]	(+++)	(++)	(-)[20,27]
**Increased thrombin time (TT)**	(++) [26,27]	(++)	ND	ND
**Late hypofibrinogenemia**	(+++) [26,27]	(++)	(-) (increased fibrinogen)	(-/+) [20,27]
**Decreased protein C activity %**	(+++) [26,27]	(+++)	ND	ND
**Thrombocytopenia**	(++) [26,27]	(++)	(++) ^e^ [20] / (+++) ^f^ [21]	(++) [20,28]

^a^: From Ebihara [27], unless otherwise noted ^b^: Infected with wild-type EBOV ^c^: Infected with MA-EBOV ^d^: Hartley guinea pigs infected with GPA-EBOV, unless otherwise noted ^e^: Inbred strain 2 United States Army Medical Research Institute for Infectious Diseases (USAMRIID) guinea pig colony infected with MA-EBOV ^f^: Inbred strain 13 USAMRIID guinea pig colony infected with GPA-EBOV ND: no data

Further, bystander lymphocyte apoptosis, an important feature in primates and mice, has not been determined in guinea pigs infected with GPA-EBOV. Mice infected with MA-EBOV differ from guinea pigs infected with GPA-EBOV and monkeys infected with wild-type EBOV in that they display a decrease in blood urea nitrogen (BUN), rather than an increase [20]. Because of these differences in EVD in rodent models, a number of therapeutic interventions that are effective in rodents challenged with adapted EBOV fail to protect NHPs challenged with wild-type EBOV from EVD (Table 2 and Table 3, see Supplemental Tables 1–2 for unabridged versions). Of the five equivalent vaccines tested in rodents and NHPs with a comparable degree of immunocompetence, two vaccines had equivalent protection in all animal models tested, and three vaccines that provided some protection in rodents were not protective in NHPs. In evaluation of peri-exposure treatment of EVD, vesicular stomatitis virus (VSV) viral vectors provided roughly the same percentage of protection in guinea pigs and NHPs. Transfer of immune serum or equivalent polyclonal or monoclonal antibodies to naïve infected animals provided no protection to NHPs and some protection to rodents. Administration of equivalent antisense phosphorodiamidate morpholino oligomers to NHPs provided less protection against EVD than rodents. 

**Table 2:** Efficacy of vaccines in animal models of Ebola virus disease.

Vaccines	Immunization Schedule	Mouse Model	Guinea Pig Model	NHP Model
***Virus Vectors***				
**HPIV3 Immunogens**	Guinea Pigs:		Complete protection with HPIV3/EBOV GP or HPIV/EBOV NP [29,31] Strong humoral response	Complete protection with 2 doses of HPIV3/EBOV GP [30] No advantage to bivalent vaccines
**EBOV GP [29,30,31]** **EBOV NP [31]** **EBOV GP + NP [30]**	IN 4 × 10^6^ PFU of HPIV3/EBOV GP [29] IN 10^5.3^ PFU of HPIV/EBOV GP or NP [31]			
	HPIV3- NHPs:			
	IN plus IT 4 × 10^6^ TCID_50_ of HPIV3/EBOV GP, HPIV3/EBOVGP+NP or 2 × 10^7^ TCID_50_ of HPIV3/EBOV GP-1–2 doses [30]			
**VSV ∆GP Immunogens**	Immunocompetent Mice:	Complete protection in NOD-SCID mice with high-dose VSV∆GP/EBOV GP [35] Complete protection with VSV∆G/EBOV GP live vector in immuncompetent mice [32,33,35] regardless of route of administration [35] Complete protection with VSV∆G/EBOV GP given 7 days prior to challenge	Complete protection with VSV ∆GP/EBOV GP [32]	67% protection with VSV ∆GP/EBOV GP in HIV+ NHPs mediated by CD4+ cells [34] Complete protection with VSV ∆GP/EBOV GP [36,37,38] in immunocompetent NHPs regardless of route of vaccination
**EBOV GP attenuated [32,33,34,35,36,37,38]**	IP, IM, IN, PO 1–2 × 10^4^ PFU of VSV∆GP/EBOV GP [32,33,35] IP 2–2 × 10^3^ PFU [35]			
	NOD-SCID Mice: IP 2 × 10^5^ PFU of VSV∆GP/EBOV GP			
	Guinea Pigs: IP 2 × 10^5^ PFU-1–2 doses of VSV∆GP/EBOV GP [32]			
	HIV + NHPs: IM 1 × 10^7^ PFU [34]			
	Immunocompetent NHPs:			
	IM 1 × 10^7^ PFU of VSV∆GP/EBOV GP [38] PO, [36] IN, [36] IM [36,37] 2 × 10^7^ PFU of VSV∆GP/EBOV GP			
**VV Immunogens**	Guinea Pigs: SC 10^7^ of VV/EBOV GP–3 doses [39]		60% protection with VV/EBOV GP [39] Survival correlated with neutralizing antibodies	No protection with VV/ EBOV GP [24] Viremia present in all subjects
**EBOV GP [24,39]**				
	NHPs: SC of VV/EBOV GP–3 doses [24]			
***Virus-like Particles (VLPs)***				
**VEEV RNA (VRP) encoding:**	Mice:	75–100% protection with VRP/EBOV NP [40,41,42] 90–100% protection with VRP/EBOV GP [41,42,43] Complete protection with VRP/EBOV GP+NP [41] 95–100% protection with VRP/EBOV VP proteins in BALB/c mice [42] 100% protection with VRP/EBOV VP 30 or VP 35 proteins in C57BL/6 mice [42] 80% protection with VRP/EBOV VP40 in C57BL/6 mice [42] No protection with VRP/EBOV VP24 protein in C57BL/6 mice [42,45]	Strain 2 guinea pigs (2 doses): no protection with VRP/EBOV NP; 60% protection with VRP-EBOV GP [41] Strain 13 guinea pigs (3 doses): complete protection with VRP-EBOV GP; 20% protection with VRP/EBOV NP 100% protection with VRP/EBOV GP [44]	No protection with VRP/EBOV GP or NP or both immunogens [24] Viremia present in all subjects Time to death similar to controls
**EBOV NP [24,40,41,42]** **EBOV GP [24,41,42,43,44]** **EBOV GP+NP [24,41]** **EBOV VP24, VP30, VP35, or VP40 [42,45]**	SC 2 x 10^6^ FFU of VRP/EBOV NP– 3 doses [40] SC 2 × 10^6^ FFU or 2 × 10^6^ IU of VRP/EBOV NP, VP24, VP30, VP35, or VP40 for 2–3 doses [42,45] SC 1 × 10^6^IU of VRP/EBOV GP or NP or GP + NP–2 doses [41] SC 1 × 10^8^ of VRP EBOV GP–4 doses [43]			
	Guinea Pigs: SC 10^7^ IU of VRP EBOV GP, NP, or GP+NP–2 or 3 doses [41,44]			
	NHPs: SC 2 x 10^6 ^FFU of VRP EBOV GP, NP or GP+NP–3 doses [24]			
***Ebola Virus Vaccines***				
**EBOV**	Mice	Complete protection with SC, IM live EBOV; [20,22,46] protection dependent on CD8+ cells and interferon-α/β receptors and not on B or CD4+ cells [20,47] Persistent infection in CD4-depleted or B cell-deficient mice [47] Complete protection with IV irradiated liposome encapsulated EBOV [49] 77% protection with IM liposome encapsulated EBOV 25, 45, or 55% protection with IP-, IM-, or IV-irradiated EBOV, respectively [48,49] >80% protection with INA-inactivated EBOV [50]		No protection with liposome encapsulated EBOV; viremia [49] 25% protection with irradiated EBOV; viremia present in all macaques [24] Neutralizing antibody titers present in 1 surviving macaque immunized with irradiated EBOV
**Live [20,22,46,47]** **irradiated [24,48,49]** **irradiated, in liposomes [24,49]** **INA+ UV irradiated, MA [50]**	SC, IM, ID 100 PFU MA-EBOV prior to IP challenge [20,22,46,47] IP 10 µg of irradiated EBOV–3 doses [48] IM, IV 1.4 µg of irradiated EBOV alone or in liposome–2 doses [49] IM 5 × 10^4 ^PFU of INA inactivated MA-EBOV–1 or 2 doses [50]			
	NHPs:			
	IV 194 µg of EBOV encapsulated in liposome–3 doses [49] SC 50 µg of irradiated EBOV–3 doses [24]			

**Abbreviations:** EBOV: Zaire ebolavirus species; FFU: focus-forming units; GP: glycoprotein; HIV: human immunodeficiency virus; GPA: guinea pig adapted; HPIV3: human parainfluenza virus type 3; IM: intramuscular; IN: intranasal; INA: 1,5-iodonaphthylazide; IP: intraperitoneal; IT: intratracheal; IV: intravenous; MA: mouse adapted; NHP: nonhuman primate; NOD: nonobese diabetic; NP: nucleoprotein; PFU: plaque-forming units; PO: oral; RNA: ribonucleic acid; SC: subcutaneous; SCID: severe combined immunodeficiency; TCID: tissue culture infective dose; VEEV: Venezuelan equine encephalitis virus; VLP: virus-like particles; VP: viral protein; VRPs: VEEV RNA replicon particles; VSV: vesicular stomatitis virus: VV: vaccinia virus

**Table 3:** Efficacy of peri-exposure treatment in animal models of Ebola virus disease.

Peri- exposure Treatment	Dose and Route of Administration	Mouse Model	Guinea Pig Model	NHP Model
***Virus Vectors***				
**VSV ∆GP Immunogens**	Mice: IP 2 x 10^5^ VSV ∆GP/EBOV GP PFU -1 day before or +30 minutes or +1 day after challenge [51]	Complete protection with VSV ∆GP/EBOV GP regardless of time of treatment [51] Mild weight loss on + 1 day, suggesting viral replication	66, 83, or 50% protection with VSV ∆GP/EBOV GP -24 or +1 or 24 hours, respectively [51]	50% protection with VSV ∆GP/EBOV GP +20–30 minutes [51]
**EBOV GP [51]**				
	Guinea pigs: IP 2 x 10^5^ VSV ∆GP/EBOV GP PFU -24 hours or +1 or 24 hours [51]			
	NHPs: IM 2 x 10^7^ PFU of VSV ∆GP/EBOV GP [51] +20–30 minutes			
***Passive Immunity***				
**Pooled immune serum to live EBOV [46,52]**	Mice: IP 1 mL of antisera (anti-EBOV IgG titers of ≥6,400) -1 day or + 1 day [46]	89% protection with pretreatment with immune serum [46] Complete protection with postchallenge treatment with immune serum Protection correlated with anti-EBOV IgG titers		No protection or delay in death with immune serum compared to controls [52] Rapid decline of anti-EBOV IgG titers by day +3 Comparable viremia in treated and control NHPs
	NHPs: IV 6 mL/kg whole blood immediately after challenge and +3 or 4 days (anti-EBOV IgG ELISA titers of 100,000) [52]			
***Passive Immunity***				
**Purified polyclonal IgG antibody against:** **Unknown, EBOV-immunized horses [53,54]**	Mice:	25% protection with horse IgG at highest dose only; lower doses not effective [54]	Complete protection with horse IgG given at day 0 only; no viremia detected [54] Complete protection with horse IgG with second dose at day +3; viremia not detected No protection if IgG is delayed until day +4; transient reduction in viremia and anti-EBOV titers not detected	No protection with horse IgG immediately postchallenge [53,54] or -2 days [54] Delayed viremia with reduction in anti-EBOV titers with NHPs receiving IgG immediately after challenge; no delay in death 33% protection with 2 doses of horse IgG
	SC 0.03, 0.3, 3 mL/kg horse IgG (log serum neutralization index of 4.2) +20–30 minutes [54]			
	Guinea Pigs: IM 1 mL/kg of horse IgG + several minutes and +3 days, or +4 days only [54]			
	NHPs:			
	IM ~1 mL/kg of horse IgG -2 days or day 0 [53,54], or day 0 and day +5 [54]			
**mAb EBOV GP-specific**	Guinea Pigs:		No protection when human mAb given +6 hours [55] 100% protection at highest dose (50 mg/kg) when human mAb given at time of challenge or -1 hour (25 mg/kg) 80% protection if human mAb given +1 hour	No protection with human mAb [56] Minimal effect on EBOV viral replication Cellular immunity may be needed for protection
**Human IgG1 [55,56]**	IP 0.5, 5, 50 mg/kg +several minutes [55] IP 25 mg/kg -1 hour, or +1 or 6 hours			
	NHPs: IV 50 mg/kg -1 day and +4 days [56]			
***Antiviral Agents***				
**Antisense Phosphorodiamidate morpholino oligomers (PMO) [57,58,59,60] **	Mice:	Complete protection with highest dose of 3 PMOs each targeting VP24, VP35, or L either pre- or postexposure [58] Complete protection following pretreatment with 500 µg (2 doses) of PMO targeting VP35 [57,58] Complete protection following pretreatment with PMOs targeting VP24 and VP35 [58,59] Postexposure protection diminishes with delay of administration of piperazine-enriched PMOs targeting VP24 and VP35 [59]	<75% protection with combination of PMOs each targeting VP24, VP35, or L given +6 days [58] <50% protection with combination PMOs given +1 day <25% protection with combination PMOs given -1 day Reduction in viral titer correlated with survival	50% protection with PMOs each targeting VP24, VP35, or L [58] High anti-EBOV antibodies and T cell responses in survivors No protection with PMO targeting VP35 only 62.5% protection with SC and IP piperazine-enriched PMOs targeting VP24 and VP35 [59] Dose dependent protection (0-60%) with IV piperazine-enriched PMOs targeting VP24 and VP35
	IP 5, [58] 50, [58] or 500 [57,58] µg of PMO targeting VP35 at -1 day and -4 hours IP 1, 5, or 50 [58,60] or 500 µg [60] of 3 PMOs targeting VP24, VP35, or L -4 hours or +1 day [58,60] IP 10 mg/kg of PMO with piperazine moieties targeting VP24 and VP35 -1 day or +1–4 days [59]			
	Guinea Pigs: IP 10 mg of each PMO targeting VP24, VP35 or L -1 day or +1 or 6 days after challenge [58]			Reduced viremia and release of IL-6 and MCP-1 with PMOs targeting VP24 and VP35
***Antiviral Agents***				
**Antisense PMOs (continued)**	s:			100 times lower viral titers in treated NHPs than in NHPs receiving control PMOs targeted to MARV proteins
	SC, IP, and IM of PMO(s) targeting VP35 or VP24, VP35, or L -2 days to through +9 days [58] SC and IP of piperazine-enriched PMOs 40 mg/kg targeting VP24 and VP35 +30–60 minutes then daily for +10 or 14 days [59] IV 4, 16, 28, or 40 mg/kg of piperazine-enriched PMOs targeting VP24 and VP35 +30–60 minutes then daily for +14 days			

**AAbbreviations:** EBOV: Zaire ebolavirus species; GP: glycoprotein; Ig: immunoglobulin; IL-6: interleukin-6; IM: intramuscular; IP: intraperitoneal; IV: intravenous; L: L polymerase; mAb: monoclonal antibody; MCP-1: monocyte chemotactic protein-1; NHP: nonhuman primate; PFU: plaque-forming units; PMO: antisense phosphorodiamidate morpholino oligomers; RNA: ribonucleic acid; SC: subcutaneous; VP: viral protein; VSV: vesicular stomatitis virus

## 4. History of Outbred Strains of Syrian Hamster in U.S.

Syrian hamsters (*Mesocricetus auratus*) are used in research studies of infectious diseases and cancer. In particular, Syrian hamsters are recognized as valuable animal models for studying emerging, acute human viral diseases caused by highly pathogenic RNA viruses. Outbred strains of Syrian hamsters in the U.S. are currently available from 3 sources: Simonsen Laboratories, Charles River Laboratories, and Harlan Laboratories. Most or all of these sources obtained Syrian hamster stock from Jerusalem. In 1930, lengthy experiments on leishmaniasis at the Hebrew University of Jerusalem were hampered by limitations of the only animal model known for the disease, the Chinese hamster (*Cricetus griseus*) [61]. Continuous replenishment of Chinese hamster stocks from China was needed as conditions for successful breeding of captive Chinese hamsters were not known, and such hamsters succumbed to a *Pasturella* epidemic in the region. Instead, an endemic species of hamster, the Syrian or golden hamster was substituted for the Chinese hamster. Syrian hamsters are easily bred in captivity, relatively free from natural hamster diseases, but are susceptible to experimental pathogens and have a short life cycle [61,62]. With the success of the domesticated Syrian hamster as a model for leishmaniasis, tuberculosis, and brucellosis, Jerusalem scientists sent shipments of Syrian hamsters to U.S. research institutions (Western Reserve University, Rockefeller Foundation, U.S. Public Health Service) in 1938 and 1942. Leishmaniasis-infected hamsters were sent to the military during World War II (probably not bred) for evaluation of therapeutic interventions [61]. U.S. research studies utilizing the Syrian stocks originally from Jerusalem were first published the 1940s [63]. In the mid 1940’s, Albert F. Marsh obtained stock probably from U.S. Public Health Service to found Gulf Hamstery [63,64]. Simonsen Laboratories acquired stock originating with Gulf Hamstery in 1962 and interbred their hamsters with stock from ARS/Sprague Dawley in 1978 [65]. In 1983, Simonsen Laboratories derived the Sim: BR golden strain that is still commercially available. Similarly, Engle Laboratory Animals and Lakeview Hamstery purchased stock from Gulf Hamstery in 1949 [63,64]. Lakeview Hamstery became a subsidiary of Charles River in 1969, and stock (Crl: LVG) is still available today [66,67]. Engle Laboratory Animals was acquired by Harlan Laboratories in 1984, and the hamster stock originally from Engle Laboratory Animals is no longer commercially available [64]. Syrian hamster stock from ARS/Sprague Dawley was sent to Central Institute for Laboratory Breeding in Hanover, Germany (HAN: AURA) in 1973 [68,69]. Harlan Laboratories purchased the Central Institute for Laboratory Breeding in 1994, and stock (HsdHan: AURA) is currently available. 

Currently available inbred strains of Syrian hamsters include Bio 1.5, Bio 14.6, Bio 15.16, Bio F1B, Bio HT, and Bio To-2 [70]. These strains are used as disease models for carcinogenicity, dental caries, cardiomyopathy, muscular dystrophy, diabetes, atherosclerosis, and hypertension.

## 5. Syrian Hamsters as a Model of Viral Hemorrhagic Fever Diseases

**Figure 1:**
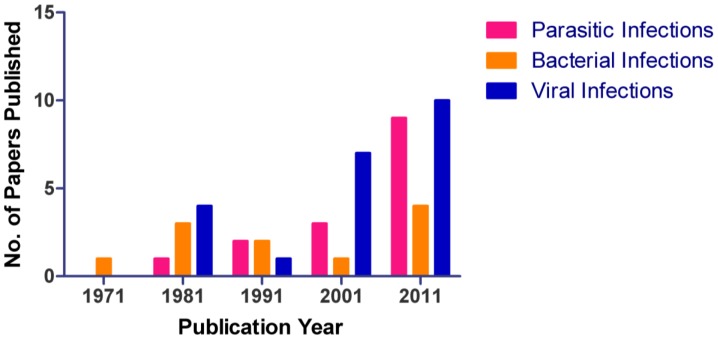
Number of publications utilizing hamsters between 1971 through 2011. An increase in use of hamsters as an animal model for parasitic, bacterial, and viral diseases is noted in the literature. During the 10-year period of 2001-2011, the largest increase in publications was observed, the majority of which were in the virology sector.

Approximately 145,895 hamsters were used in research in 2010 in the U.S. comprising 13% of total animal usage [71]. Although hamsters are still widely used, the number of hamsters currently used in research is well below peak usage of over 500,000 in the 1980s [72]. However, the total number of papers published from 1971 to 2011 has steadily increased (Figure 1) with the greatest increase in studies of viral infections. Availability, size, ease of care and breeding in laboratory conditions, and cost contribute to the popularity of Syrian hamster as an alternative to nonhuman primates for viral research.

Hamsters are valuable animal models for studying viral hemorrhagic fevers, including EVD [27], Marburg virus disease [73], hantavirus cardio-pulmonary syndrome [74,75,76,77,78,79], arenavirus hemorrhagic fevers [80], yellow fever hemorrhagic fever [80,81,82], and phlebovirus models of Rift Valley fever (Table 4) [83]. All of these viruses cause hemorrhagic fevers in hamsters, but infections caused by some of these viruses (e.g., Pirital, Maporal) that serve as disease models (e.g., hantavirus cardiopulmonary syndrome, Lassa fever) are not pathogenic in humans [78,84]. A number of these viruses were hamster adapted (Marburg, yellow fever, Pichinde) [73,80,81,85] or mouse adapted (Ebola) [27].

**Table 4:** Hamsters as a model for viral hemorrhagic fevers.

Pathology and laboratory abnormalities	Virus
***Spleen***	
Necrosis of white and red pulp, lymphoid zone	Punta Toro [83]
	Pirital [84,86,87]
	Marburg [73]
Mild lymphoid depletion of white pulp, early infection	Ebola [27]
Lymphoid necrosis and reactive hyperplasia	Gabek Forest [83]
	Yellow fever [81,82,85]
Destruction of tissue architecture, terminal phase	Ebola [27]
Mononuclear infiltrate expanding red pulp and obscuring lymphoid architecture	Andes [75]
Proliferation of reticuloendothelial tissue, macrophages in sinuses	Marburg [73]
Apoptosis of mononuclear phagocytic system and lymphocytes, terminal phase	Ebola [27]
***Liver***	
Hepatocellular necrosis	Punta Toro [83]
Hepatocellular necrosis, hemorrhage and fibrin deposition, inflammation	Ebola [27]
Hepatocelullar necrosis, mild steatosis	Gabek Forest [83]
Lobular microvesicular steatosis, monocytic infiltration, necrosis	Yellow fever [81,82,85]
Apoptosis/necrosis with inflammatory infiltration	Pirital [84,86,87]
	Andes [75,76,79]
Interstitial mononuclear infiltration	Maporal [78]
Increased AST	Yellow Fever [81,85]
	Pirital [84,86]
Increased total bilirubin	Yellow Fever [81]
Increased ALT	Punta Toro [83]
	Gabek Forest [83]
	Yellow Fever [81,85]
	Pichinde [80]
	Pirital [84,86]
***Lymph nodes***	
Lymphoid necrosis and reactive hyperplasia	Punta Toro [83]
Lymphoid necrosis and reactive hyperplasia, late infection	Ebola [27]
Lymphoid depletion and sinus hemorrhage, terminal phase	Ebola [27]
Follicular and plasma cell hyperplasia	Andes [76]
Proliferation of reticuloendothelial tissue, macrophages in sinuses	Marburg [73]
Histiocytosis and neutrophilia, early infection	Ebola [27]
Apoptosis of macrophages, dendritic cells, late infection	Ebola [27]
***Lung***	
Alveolar hemorrhage with histiocytic infiltration	Yellow fever[81]
Alveolar edema, fibrin deposition, pleural effusion	Andes[75,79]
	Maporal [78]
Interstitial pneumonitis, diffuse or focal atelectasis, hemorrhagic necrosis	Punta Toro [83]
	Pirital [87]
Interstitial pneumonitis, hemorrhage	Gabek Fores [83]
	Pirital [84,86]
	Andes [76]
Interstitial pneumonitis, proliferation of vascular endothelium, capillary congestion, fibrin deposition	Marburg [73]
***Kidney***	
Tubular necrosis	Yellow Fever [82,85]
Tubular epithelium degeneration, mononuclear cell infiltration, intracytoplasmic bodies, thickening of Bowman’s capsule, shrinkage of glomerular tufts	Marburg [73]
Glomerular necrosis	Gabek Forest [83]
Interstitial nephritis	Maporal [78]
Increased creatinine, blood urea nitrogen concentrations	Pirital [84,86]
***Vascular dysregulation***	
Vascular leakage (edema, effusion)	Andes [75,79]
	Pichinde [80]
	Yellow fever [80]
	Maporal [78]
Decreased albumin concentrations	Pichinde [80]
	Yellow fever [81]
	Pirital [86]
***Coagulopathy***	
Increased PT, aPTT	Yellow Fever [81]
	Pirital [84,86]
	Andes [76]
	Ebola [27]
Increased TT	Pirital [86]
	Ebola [27]
Early increased, then decreased fibrinogen concentrations Decreased fibrinogen concentrations, late infection Increased fibrinogen concentration	Yellow fever [81]
	Ebola [27]
	Andes [76]
	Pirital [84]
Increased D-dimer concentrations	Pirital [84]
Decreased protein C concentrations	Ebola [27]
Decreased protein S concentrations, late infection	Andes [76]
Thrombocytosis, mid or late infection	Pirital [84,86]
Thrombocytopenia, late infection	Andes [75]
	Yellow fever [81]
***Hematological abnormalities***	
Early leukopenia then leukocytosis (primarily neutrophils)	Yellow fever [81]
Leukocytosis mid-to-late infection	Pichinde [80]
	Pirital [86]
	Andes [75]
Lymphopenia	Andes [75]
***Cytokines***	
Increased blood cytokine concentrations, cross reactive mice antibodies	Pichinde [80]
	Andes [76]
Increased gene expression of cytokines	Andes [76]
	Yellow fever [85]
	Ebola [27]

Viral hemorrhagic fever is a syndrome characterized by fever, malaise, increased vascular permeability, and coagulation abnormalities that may lead to hemorrhage [80,88]. A number of factors contribute to alterations in vascular function such as direct cytolytic infection of the endothelium, changes in tight junctions between endothelial cells, alterations in coagulation pathways, disruption of hematopoiesis, and/or the release of cytokines and other permeability factors (e.g., tissue factor, TNF-α, nitric oxide) from endothelial cells, neutrophils, macrophages, and/or monocytes [1,80,89,90,91,92]. Although EBOV does infect endothelial cells of NHPs, infection occurs late in the disease course after the development of disseminated intravascular coagulation [89]. Rather, researchers consider the release of cytokines and other vasoactive mediators to disrupt the endothelial barrier, causing plasma volume loss, hypovolemic shock, multi-organ failure, and death [93]. Impairment of liver function may alter the production of vitamin K-associated coagulation factors (e.g., factor VII) and coagulation inhibitors (e.g., protein C) that could contribute to coagulopathy [26,27,92]. Thrombocytopenia occurs in part due to consumptive coagulopathy, but evidence from bone marrow aspirates in EBOV-infected nonhuman primates also reveals damaged megakaryocytes and atypical platelets [91]. 

Among the Syrian hamster models of viral hemorrhagic fevers, liver and lungs are the commonly affected organs. Signs of tachycardia and tachypnea and results of hematological, blood chemistry, and coagulation tests indicative of vascular leakage or shock are noted in Syrian hamsters infected with Andes virus, [77] Pichindé or Pirital viruses, [80,84,86] or yellow fever virus [80]. Upregulation of cytokines in one study of hamsters challenged with Pichindé virus preceded vascular leakage [80]. The search for an animal model that more closely resembles human EVD than other rodent models to date led to the development of the Syrian hamster model.

## 6. Syrian Hamsters as an Ebola Virus Disease Model

**Figure 2:**
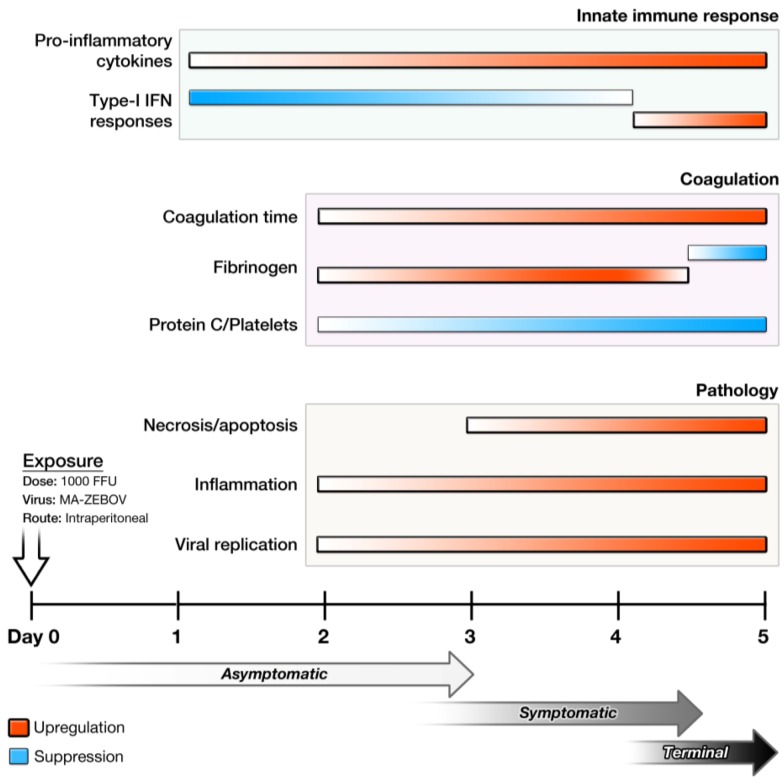
Temporal progression of disease in the Syrian hamster model of Ebola virus disease. Following exposure to 1000 focus-forming units of MA-EBOV IP, hamsters begin showing signs of illness around day 3. Changes in the innate immune response, coagulation parameters, and pathology are observed as early as days 1 and 2.

Data from a study of Syrian hamsters challenged IP or SC with MA-EBOV or wild-type EBOV indicate that only MA-EBOV given IP causes EVD reminiscent of human disease including, severe coagulopathy, lymphocyte apoptosis, cytokine dysregulation (e.g., suppression of early type I IFN responses), target organ necrosis and/or apoptosis (*i.e*., lymph nodes, spleen, liver), and lethal outcome (Table 5, Table 6) (Figure 2) [27]. Such suppression/non-induction of type-I IFN response and aberrant pro-inflammatory responses are suggested as some of the key pathogenic processes that lead to lethal outcome [20,27]. In contrast to MA-EBOV challenge, wild-type EBOV given IP in hamsters causes activation of early type-I IFN responses, mild inflammatory responses, induction of less-prominent apoptosis, and minimal pathological changes [27].

**Table 5:** Comparison of current animal models of Ebola virus disease.

	Macaque	Guinea pig	Hamster	Mouse
***Hallmarks of Human Disease***				
Target cells/organs	+	+	+	+
Cytokine dysregulation	+	ND ^a^	+	+/-
Lymphocyte apoptosis	+	ND ^a^	+	+
Coagulation dysfunction	++	+	++	+/-
***Advantages/Disadvantages***				
Availability	-	+/-	+	+
Ease of handling	-	+/-	+	+
Research reagents	++	-	+/++	+++
Pathogenicity of MA-EBOV	++ [20] ^b^	+	+++	+++

^a^: ND = no data ^b^: MA-EBOV = mouse-adapted EBOV

**Table 6:** Comparison of pathological changes in different animal models of Ebola virus disease.

	Liver			Spleen		
	Hepatocellular degeneration and necrosis	Inflammation	Fibrin	Lymphoid necrosis and loss	Inflammation	Fibrin
**Mouse**	Diffuse, random	Neutrophilic	Little	Multifocal, mild	Neutrophilic	Little
**Guinea pig**	Diffuse, random	Neutrophilic and histiocytic	Little to moderate	Diffuse, severe	Neutrophilic	Little to moderate
**Hamster**	Diffuse, midzonal	Neutrophilic	Little	Diffuse, moderate to severe	Neutrophilic	Little
**NHP**	Diffuse, random	Neutrophilic	Abundant	Diffuse, severe	Neutrophilic	Abundant

**Figure 3:**
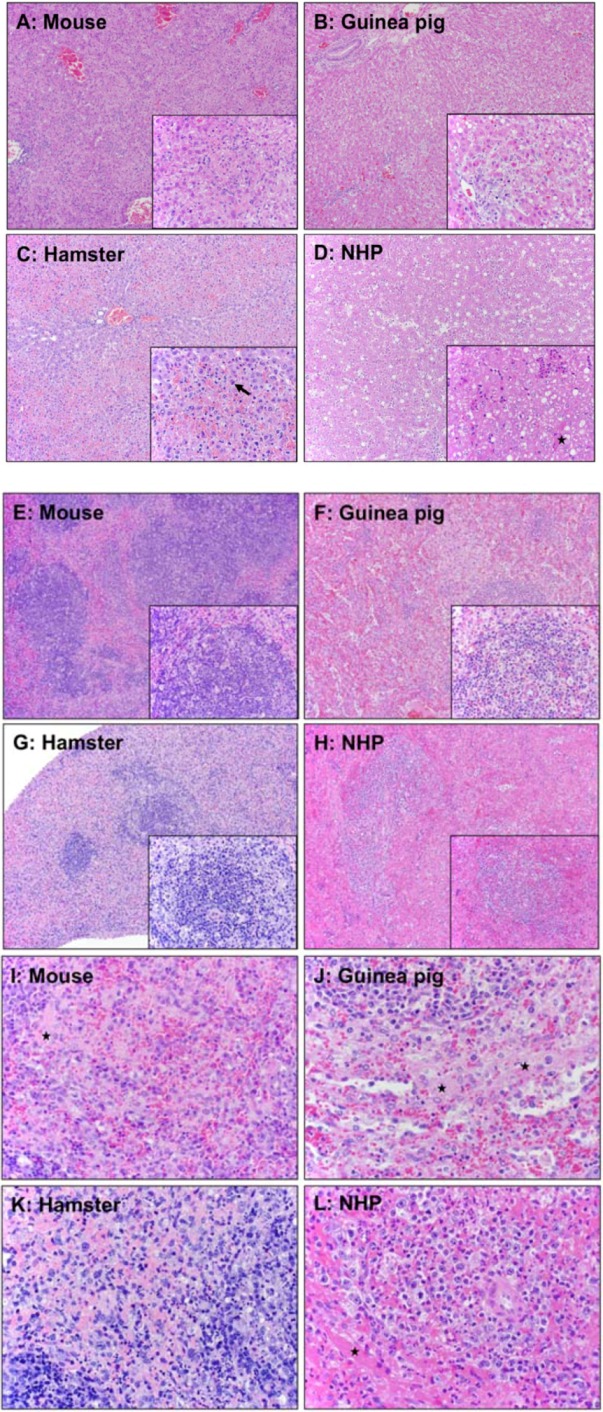
Comparison of pathology in mouse, guinea pig, hamster, and nonhuman primate. A Balb/c mouse and a Syrian hamster were infected IP with MA-EBOV; a Hartley strain of guinea pig was infected with GPA-EBOV; and a macaque was infected with wild-type EBOV. **(A-D)** Pathological changes in liver of different animal models. (**A**) Mouse: Multifocal, random hepatocellular degeneration and necrosis (10x and 40x inset). **(B**) Guinea pig: Diffuse, random hepatocellular degeneration and necrosis. Inflammatory cells are nearly absent (10x and 40x inset). (**C–D**) Hamsters: Liver. (**C**) Diffuse, midzonal hepatocellular degeneration, necrosis, and congestion. Inflammatory cells are nearly absent (10x). Solid arrow: prominent intracytoplasmic filovirus inclusion bodies in hepatocytes (40x). (**D**) Diffuse, random hepatocellular degeneration and necrosis (10x). Solid star: fibrin deposition (40x inset). (**E-L**) Pathological changes in spleen of different animal models. (**E** and **I**) Mouse: White and red pulp. White pulp (**E**); diffuse lymphoid necrosis and loss (10x and 40x inset). Red pulp (**I**); mild to moderate acute splenitis and small amounts of fibrin (solid star) (40x). (**F** and **J**) Guinea pig: White and red pulp. White pulp **(F**); multifocal lymphoid necrosis (10x and 40x inset). Red pulp (**J**); multifocal, mild to moderate acute splenitis with necrosis. Solid star: small amounts of fibrin at marginal zone (40x). (**G** and **K**) Hamster: White and red pulp. White pulp **(G)**; diffuse lymphoid necrosis (10x and 40x inset). Red pulp (**K**); mild to moderate acute splenitis with monocytic degeneration and necrosis (40x). (**H** and **L**) NHP: White and red pulp. White pulp (**H**); diffuse lymphoid necrosis (10x and 20x inset). Red pulp (**L**); diffuse, moderate acute splenitis (40x). Solid star: fibrin.

The severity of coagulopathy observed in Syrian hamsters infected with MA-EBOV is similar to that observed in rhesus macaques following challenge with wild-type EBOV (Table 1). Hepatic changes in Syrian hamsters closely resemble those of rhesus macaques, including disseminated hepatocellular degeneration and necrosis with infiltration of moderate numbers of neutrophils and fewer macrophages than neutrophils (Table 6). In contrast to macaques, little fibrin deposition occurs within hepatic sinusoids of hamsters (Figure 3). Likewise, splenic lesions in hamsters are also similar to those observed in macaques and are characterized by necrosis of lymphocytes and marked loss of white pulp. Additionally, multifocal acute splenitis is characterized by moderate numbers of viable and degenerate neutrophils and fewer macrophages than neutrophils mixed with necrotic debris within the red pulp. Lymph nodes also display diffuse lymphoid necrosis and loss along with acute lymphadenitis and draining hemorrhage (Figure 3). In terminal Syrian hamsters, all cytokines tested (IL-1β, IL-2, IL-4, IL-6, and IL-12p35; tumor growth factor [TGF]-β; IFN-γ induced protein [IP]-10 and IFN-γ; TNF-α) are upregulated in the spleen, liver, and blood, indicating potentially uncontrolled immune responses. 

## 7. Reagent and Assay Development

Until recently, lack of available reagents and specific assays to monitor host responses in hamsters (including early innate immune responses) limited investigators to studies on disease progression, humoral immune responses, and pathology. The lack of a complete genome sequence of the Syrian hamster has retarded the development of molecular, genetic, and antibody-based assays. In lieu of a complete genome, a number of studies evaluated the cross reactivity of antibody-based (ELISA, Luminex^®^) assays developed for other rodents against Syrian hamster cytokines, chemokines, adherins, growth factors, and other serum factors (Table 7) [76,80,94]. Data from most of these studies indicate limited cross reactivity of Syrian hamster proteins to other rodent antibodies. However, monoclonal antibodies from rats, mice, goats, and rabbits specific for hamsters successfully identified hamster surface markers of immune cells (T cells, B cells, dendritic cells, macrophages) via flow cytometry [74,95,96,97]. Microarray proteome expression studies have quantified hamster responses to disease through cross species hybridization of Syrian hamster RNA to cDNA from other species (e.g., rat, mouse, human) [98,99,100,101,102]. The complete transcriptome of the Syrian hamster has been determined but is not yet publicly available, and a microarray chip is currently under development. As hamster-specific antibodies have not been made against cytokines/chemokines, gene expression of these factors during infection is tracked through quantitative reverse transcriptase polymerase chain reaction (qRT-PCR) [103,104,105,106,107,108,109]. Recently, use of qRT-PCR has been extended to include 51 registered hamster gene sequences targeting apoptosis, cell junction integrity, cell proliferation, and coagulation in addition to immunological responses [94]. qRT-PCR assays were utilized to profile host responses in hamsters infected with yellow fever virus, Andes virus, and EBOV [27,85,94,109]. Use of qRT-PCR assays will also contribute to identification of host response factors needed for survival in animals treated with antiviral drugs and of protective immune response in vaccinated animals. Such assays will be used until the full genome sequence is available for the development of large scale microarrays.

**Table 7:** Cross-reactive or hamster-specific reagents.

**Cross-reactive antibodies **	
Mouse anti-mouse/rat MHC II [74]	Rat anti-mouse CD4 [74,96,97]
Mouse anti-mouse Thy1.2 [74]	
Mouse anti-rat CD8, CD8β [74,97]	
**Mouse, rabbit, and goat anti-hamster antibodies**	
Mouse dendritic cell marker [95]	Mouse CD18 [95]
Mouse pan lymphocyte [95]	Mouse MHC II [95]
Mouse T cell [95]	Rabbit IgG [95]
Mouse B cell [95]	Rabbit IgM [74]
Mouse CD45 [95]	Goat IgG [74,78,96,97]
**Cross-reactive cytokine, chemokine, and serum factor assays **	
Rat GM-CSF [94]	Mouse / Rat VCAM-1 [76,80]
Rat Leptin [94]	Mouse / Rat vWF [80]
Rat GRO/KC [94]	Mouse / Rat VEGF [80]
Rat / Mouse IL-1α [80,94]	Mouse / Rat MDC [80]
Mouse MIG [94]	Mouse / Rat SCF [80]
Mouse IL-13 [94]	Mouse GCP-2 [80]
Mouse / Rat IP-10 [76,80]	Mouse MCP-3 [80]
Mouse /Rat M-CSF [76,80]	Mouse MIP-2 [80]
Mouse /Rat MCP-1 [76,80]	Mouse MIP-3β [80]
Mouse Fibrinogen [80]	Mouse AST [80]
**Cross-reactive microarray hybridization **	
DNA Microarrays	MicroRNA Microarrays
Rat Genome [98,102]	Human [99] Rat Mouse
Mouse genes [100,101]	

## 8. Future Perspectives

Although the predictive value of the hamster model for efficacy testing of vaccines and therapeutics remains to be shown, numerous research tools are now available that will facilitate the use of this animal model in future research on Ebola virus pathogenesis. The newly developed hamster EVD model will certainly augment and perhaps may one day replace mice and guinea models as an alternative model for pathogenesis studies and efficacy testing. Hamsters infected with MA-EBOV currently exhibit EVD manifestations most similar to primates, particularly with respect to coagulation abnormalities. Of particular interest is employment of this hamster model to confirm the efficacy of drugs used in NHPs to control coagulopathy (e.g., recombinant activated protein C, recombinant nematode anticoagulant protein c2). 

## Supplementary Files

Supplementary File 1
