# Supplementary material for: Use of the Syrian Hamster as a New Model of Ebola Virus Disease and Other Viral Hemorrhagic Fevers
Source: Viruses. 2012 Dec 14;4(12):3754–84. doi: 10.3390/v4123754 (PMC3528289; doi:10.3390/v4123754)
Supplement: Supplementary File 1 [file viruses-04-03754-s001.pdf]

Supplemental Table 1. Efficacy of vaccines in animal models of Ebolavirus disease.

Vaccines	Immunization Schedule	Mouse Model	Guinea Pig Model	NHP Model
Virus Vectors				
**HPIV3 Immunogens**<br>• **HPIV3 ΔHN-F/ EBOV GP [1]**<br>• **EBOV GP [1-3]**<br>• **EBOV NP [2]**<br>• **EBOV GP + NP [3]**<br>• **EBOV GP +GM-CSF [3]**	Guinea Pigs:<br>• IN 4 x 10^6 PFU of HPIV3 ΔHN-F/EBOV GP or HPIV3/EBOV GP [1]<br>• IN $10^{5.3}$ PFU of HPIV/EBOV GP or NP [2]<br>HPIV3- NHPs:<br>• IN plus IT 4 x 10^6 $TCID_{50}$ of HPIV3/EBOV GP, HPIV3/EBOV GP+GM-CSF, HPIV3/EBOVGP NP or 2 x 10^7 $TCID_{50}$ of HPIV3/EBOV GP for 1–2 doses [3]		• Complete protection with HPIV3 ΔHN-F/EBOV GP, HPIV3/EBOV GP, or HPIV3/EBOV NP [1, 2]<br>• Strong humoral response	• Complete protection with 2 doses of HPIV3/EBOV GP [3]<br>• No advantage to bivalent vaccines
**RABV ΔGP/EBOV GP**<br>• **(Live attenuated) [4]**<br>• **RABV/EBOV GP fused to GCD of RABV (inactivated) [4]**	Mice: IM 5 x 10^5 FFU	• Complete protection with either vector<br>• EBOV GP incorporation into virions not dependent on RABV GCD		
**Human Ad5 Immunogens**<br>• **CMVEBOV GP [5-9]**<br>• **CAGoptEBOV GP [8, 9]**	Mice:<br>• IN, PO, IM 1 x 10^{10} [6] to 5 x 10^{10} [5] particles of Ad5/CMVEBOV GP<br>• IP 1 x 10^8 PFU Ad5/CMVEBOV GP[7]<br>• IM 1 x 10^4–1 x 10^7 IFU of Ad5/CMVEBOV GP or 1 x 10^4–1 x 10^6 IFU of Ad5/CAGopt EBOV GP [9]<br>Guinea Pigs:<br>• IN, IM 1 x 10^{10} particles of	• With induced preexisting Ad5 immunity, complete protection with only IN Ad5/CMVEBOV GP [5]<br>• With no Ad5 immunity: complete protection regardless of route [5-7, 9]<br>• Mucosal immunization Ad5-EBOV GP increased cellular and humoral immunity compared to IM immunization[6]	• With systemically induced preexisting Ad5 immunity, complete protection with IN Ad5/CMVEBOV GP [8]<br>• With mucosally induced preexisting Ad5 immunity, 83% protection with IN Ad5/CMVEBOV GP<br>• With systemically induced preexisting Ad5	

Vaccines	Immunization Schedule	Mouse Model	Guinea Pig Model	NHP Model
	Ad5/CMVEBOV GP or Ad5/CAGopt EBOV GP[8]	• Complete protection with Ad5/CAGopt EBOV GP and 2 higher doses of Ad5/CMVEBOV GP [9]<br>• Increased cellular and humoral responses with Ad5/CAGoptEBOV GP	immunity, 78% or 100% protection with IM or IN CAGopt EBOV GP, respectively<br>• With no Ad5 induced immunity, complete protection regardless of route	
**AdC7/CMVEBOV GP[10]**	Mice: IM 5 x 10^9–5 x 10^{10} particles of AdC7/EBOV GP<br>Guinea Pigs: IM 5 x 10^9–5 x 10^{11} particles/kg	• Complete protection; not affected by induced preexisting Ad5 immunity	• Complete protection<br>• Effect of preexisting Ad5 immunity not studied	
**AdC5/1-CMVEBOV GP [11]**	Mice: IM 5 x 10^{10} particles	• Complete protection		
**VSV ΔGP Immunogens**<br>• **EBOV GP attenuated [12-18] or irradiated[13]**<br>• **TAFV GP [12]**<br>• **RESTV GP**<br>• **SUDV GP**<br>• **SUDV GP+VP40**<br>• **SUDV GP + NP**<br>• **SUDV GP+NP and SUDV GP+VP40**	Immunocompetent Mice:<br>• IP, IM, IN, PO 1–2 x 10^4 PFU of VSVΔG/EBOV GP, TAFV GP, RESTV GP, or SUDV GP [12, 13, 15]<br>• IP 2–2 x 10^3 PFU of VSVΔG/EBOV GP [15]<br>NOD-SCID Mice: IP 2 x 10^5 PFU of VSVΔG/EBOV GP<br>Guinea Pigs: IP 2 x 10^5 PFU of VSVΔG/EBOV GP, TAFV GP, RESTV GP, SUDV GP, SUDV GP+VP40, SUDV GP+NP, SUDV GP+NP and SUDV GP+VP40 for 1–2 doses [12]<br>HIV+ NHPs: IM 1 x 10^7 PFU of VSVΔG/EBOV GP [14]<br>Immunocompetent NHPs:<br>• IM 1 x 10^7 PFU of	• Complete protection with VSVΔG/EBOV GP live vector in immunocompetent mice [12, 13, 15]regardless of route of administration [15]<br>• Complete protection with VSVΔG/EBOV GP given 7 days prior to challenge<br>• No protection with irradiated vaccine [13]<br>• Complete cross-EBOV species protection with VSVΔGP/RESTV GP or VSVΔGP/TAFV GP [12]<br>• 75% cross-EBOV species protection with VSVΔG/SUDV GP [12]<br>• Complete protection in NOD-SCID mice with high-dose VSVΔGP/EBOV GP [15]	• Complete protection with homologous VSV ΔGP/EBOV GP only [12]<br>• 83% cross EBOV species protection with 2 doses of VSV ΔGP/SUDV GP+VP40<br>• IgG antibodies against both SUDV antigens not increased after EBOV challenge indicating lack of viral replication<br>• No EBOV neutralization antibodies detected following SUDV vaccination and EBOV challenge<br>• Reduced cross-EBOV species protection with SUDV NP inclusion	• 67% protection with VSV ΔGP/EBOV GP in HIV+ NHPs mediated by CD4+ cells [14]<br>• Complete protection with homologous PO,[16] IN, [16]OR IM [16, 17] VSV ΔGP/EBOV GP to IM [16, 18] or aerosol [17] EBOV challenge<br>• 25% protection with VSV ΔGP/EBOV GP following rechallenge with SUDV [18]

Vaccines	Immunization Schedule	Mouse Model	Guinea Pig Model	NHP Model
	VSVΔGP/EBOV GP then EBOV challenge and SUDV rechallenge [18]<br>• PO,[16] IN,[16] IM[16, 17] 2 x 10^7 PFU of VSVΔGP/EBOV GP			
**CMV Δm157/EBOV NP_{CTL}[19]**	Mice: IP 5 x 10^5 PFU–2 doses	• Complete protection		
**VV Immunogens**<br>• **EBOV NP[20]**<br>• **EBOV VP35**<br>• **EBOV VP40**<br>• **EBOV GP [20, 21]**<br>• **EBOV sGP [20]**	Guinea Pigs: SC 10^7 of VV/EBOV NP, VP35, VP40, GP, OR sGP–3 doses[20]<br>NHPs: SC of VV/EBOV GP–3 doses[21]		• 60% protection with VV/EBOV GP only [20]<br>• Survival correlated with development of neutralizing antibodies	• No protection with VV/EBOV GP [21]<br>• Viremia present in all subjects<br>• Time to death similar to controls
Virus-like Particles (VLPs)				
**VEEV RNA replicon particles (VRP encoding:**<br>• **EBOV NP [21-24]**<br>• **EBOV GP [21, 23-26]**<br>• **EBOV GP +NP[21, 23]**<br>• **EBOV VP24 [24, 27]**<br>• **EBOV VP30**<br>• **EBOV VP35**<br>• **EBOV VP40**<br>• **EBOV GP + Lassa GP combination [26]**<br>• **EBOV GP/Lassa GP bivalent**	Mice:<br>• SC 2 x 10^6 FFU of VRP/EBOV NP3 doses [22]<br>• SC 2 x 10^6 FFU or 2 x 10^6 IU of VRP/EBOV NP, VP24, VP30, VP35, or VP40 for 2-3 doses [24, 27]<br>• SC 1x10^6 IU of VRP/EBOV GP, NP, or GP+NP for 2 doses [23]<br>• SC 1 x 10^8 of VRP EBOV GP- 4 doses [25]<br>Guinea Pigs:<br>• SC 10^7 IU of VRP EBOV GP, NP, or GP+NP–2 or 3 doses [23]<br>• SC 10^7 IU of VRP EBOV	• 75-100% protection with VRP/EBOV NP [22-24]<br>• 90–100% protection with VRP/EBOV GP [23-25]<br>• Complete protection with VRP/EBOV GP+NP [23]<br>• 95–100% with VRP/EBOV VP proteins in BALB/c mice[24]<br>• 100% protection with VRP/EBOV VP 30 or VP 35 proteins in C57BL/6 mice [24]<br>• 80% protection with VRP/EBOV VP40 in C57BL/6 mice [24]<br>• No protection with VRP/EBOV VP24 proteins in C57BL/6 mice [24, 27]	• Strain 2 guinea pigs (2 doses): no protection with VRP-EBOV NP; 60% protection with VRP-EBOV GP [23]<br>• Strain 13 guinea pigs (3 doses): complete protection with VRP-EBOV NP+GP or VRP-EBOV GP; 20% protection with VRP-EBOV NP<br>• 80% protection with bivalent VRP/EBOV GP with Lassa GP[26]<br>• 100% protection with VRP/EBOV GP and VRP/Lassa GP	• No protection with VRP/EBOV GP or NP or both immunogens [21]<br>• Viremia present in all subjects<br>• Time to death similar to controls

Vaccines	Immunization Schedule	Mouse Model	Guinea Pig Model	NHP Model
	GP+Lassa GP-3 doses [26]<br>NHPs: SC 2 x 10^6 FFU of VRP EBOV GP, NP or GP+NP–3 doses [21]			
**KUN replicon encoding:**<br>• **EBOV GP [28]**<br>• **EBOV GP/Ctr**<br>• **EBOV GP mutant for ease of shedding (D637L)**	Guinea Pigs: IP 1 x 10^6 or 5 x 10^6 VLPs-2 doses		• >75% protection with KUN/GP or KUN/GP mutant at higher dosage<br>• 50% protection with KUN/GP mutant at lower dosage<br>• 25% protection with KUN/GP at lower dosage<br>• No protection with KUN/soluble GP	
**rBV encoding:**<br>• **EBOV VP40**<br>• **EBOV GP [29]**<br>**rBV replicon encoding:**<br>• **EBOV VP40**<br>• **EBOV GP**<br>• **EBOV NP [30]**<br>**293T cell-derived EBOV VP40 + GP + NP [30]**	Mice:<br>• IM 1, 10 or 50 ug of rBV or 293T cell derived/EBOV VP40 + GP + NP VLPs–2 doses [30]<br>• IM 10 or 50 µg of rBV/EBOV VP40 + GP VLPs–2 doses [29]<br>• IM 10 ug of rBV/EBOV VP40 GP VLPs–3 doses	• Dose dependent protection with 2-dose regimen; complete protection at highest dose [29, 30]<br>• 83% protection with 3 doses[29]<br>• Equivalent immune responses and protection from challenge with rBV-derived or 293T cell-derived VLPs[30]		
**Liposomes encapsulating:**<br>• **EBOV GP + VP40 [31-33]**<br>• **EBOV NP+GP+VP40 [34, 35]**<br>• **EBOV GP + MARV VP40 [31]**<br>• **EBOV VP40 +MARV GP**	Mice: IM, IP 0.1, 1, or 10 µg EBOV GP + VP40 VLPs for 2 [33] or 3 doses [32]<br>STAT-1 KO mice: IM 10 µg EBOV GP + VP40 + NP–3 doses [34]<br>Guinea Pigs:	• Complete or nearly complete protection with highest dosage EBOV GP + VP40 VLPs in BALB/c,[32] C57Bl/6,or perforin-deficient mice [33]<br>• 50% protection in CD4+-	• Complete protection with EBOV GP + VP40 VLPs [31]<br>• 90% protection with EBOV GP + MARV VP40 VLPs or equal mixture of EBOV GP + VP40 and MARV GP + VP40 VLPs	• Complete protection with EBOV NP+GP+VP40 [35]<br>• Viremia and clinical or laboratory signs of EBOV infection not detected in vaccinated

Vaccines	Immunization Schedule	Mouse Model	Guinea Pig Model	NHP Model
• **MARV GP + VP40**	• IM 100 µg of EBOV GP + VP40 VLPs [31]<br>• IM 50 µg EBOV GP + VP40 and 50 µg MARV GP + VP40 VLPs<br>NHPs: IM 250 µg EBOV NP + GP + VP40 VLPs in RIBI adjuvant–3 doses [35]	deficient mice<br>• 13% protection in IFN-γ-deficient mice<br>• No protection in B cell-, βδ TCR-, CD8+-, or STAT-1-deficient mice[33, 34]	• No protection with EBOV VP40 +MARV GP<br>• High antibody titers and no viremia in survivors	and challenged NHPs<br>• Strong antibody and T cells (tumor necrosis factor-α) responses
DNA Vaccines				
**DNA Plasmid Immunogens**<br>• **EBOV GP [36-40]**<br>• **EBOV GP glycosylation deletions[37]**<br>• **EBOV sGP[39]**<br>• **EBOV NP [36, 39, 40]**<br>• **EBOV GP + NP [38]**<br>• **EBOV GP + TAFV GP+ SUDV GP + EBOV NP**<br>• **EBOV GP, Marv GP, VEEV 26s, Anthrax PA [40]**	Mice:<br>• Prime-0.5 µg of EBOV GP DNA, then 3 boosts with 1.5 µg via gene gun [36]<br>• 0.5 or 3 µg of EBOV NP or GP DNA via gene gun–3 doses [36]<br>• 0.25–0.5 ug EBOV GP and glycosylation mutants DNA via gene gun–3 doses [37]<br>• 5 ug EBOV GP or NP DNA via gene gun-2 doses [40]<br>Guinea Pigs:<br>• 5 µg of EBOV GP DNA via gene gun-3 doses [40]<br>• 5 µg each of EBOV GP, MARV GP, VEEV 26s, Anthrax PA DNA via gene gun–3doses[40]<br>• IM 100 µg of EBOV GP DNA–3 doses [38]<br>• IM 75 µg of EBOV GP DNA + 25 µg of EBOV NP DNA–3 doses [38]	• Complete protection with EBOV GP DNA prime/boost [36]<br>• Similar dose-dependent partial protection (~60–90%) with either EBOV NP or GP DNA with boosts[36]<br>• 89% protection with EBOV GP DNA [37]<br>• 29–31% protection with deletion of mucin region or an N-linked GP2 glycosylation site involved in dimerization of GP1 and GP2<br>• IgG antibody titer generally correlated with protection with EBOV GP or glycosylation mutants DNA<br>• Complete protection with 2 doses of EBOV GP or NP DNA; high antibody response[40]	• Complete protection with EBOV GP DNA alone[38, 39] or in combination with NP, or EBOV NP in combination with TAFV GP, EBOV GP SUDV GP and EBOV GP DNA[38]<br>• Complete protection with EBOV NP or GP DNA and 83% protection with EBOV sGP DNA if challenge was within 2 months following first immunization [39]<br>• Lower protection and antibody titers to immunogens if time to challenge nearly doubles<br>• 67% protection with EBOV GP DNA[40]<br>• 60% protection with multivalent EBOV GP, Marv GP, VEEV 26s, Anthrax PA DNA	

Vaccines	Immunization Schedule	Mouse Model	Guinea Pig Model	NHP Model
	• IM 25 μg each of DNA plasmids encoding EBOV GP, TAFV GP, SUDV GP, and EBOV NP–3 doses<br>• IM 50 μg of EBOV GP, sGP, or NP DNA–4 doses [39]			
**DNA Prime, rBV boost[41]**<br>• **EBOV GP**<br>• **EBOV GP/Ctr**	Guinea Pigs:<br>• Prime: 2.5 μg EBOV GP DNA via gene gun, then 2 boosts: SC 5 μg of rBV-derived EBOV GP or GP/Ctr<br>• Prime and 2 boosts of rBV EBOV GP or GP/Ctr or EBOV GP DNA		• 50% protection with prime and boosts of rBV EBOV GP<br>• 33% protection with DNA prime, rBV EBOV GP/Ctr boosts<br>• 17% protection with prime and boosts of EBOV GP DNA or rBV EBOV GP/Ctr<br>• No protection with prime with EBOV GP DNA and boosts with rBV EBOV GP	
Fusion Proteins				
**RESTV GP1 fused to GP1 mAb forming immune complex [25]**	Mice:<br>• SC 10 μg of immune complex alone or with PIC and/or alum adjuvant–4 doses<br>• SC 10 or 25 μg of immune complex with adjuvant–3 doses	• 80% protection with 4 doses of immune complex and PIC with or without alum adjuvant<br>• 50% protection with 3 doses of immune complex (25 μg) plus both adjuvants<br>• 20% protection with3 doses of immune complex (10 μg) plus both adjuvants		
**RESTV GP fused to Fc IgG1 fragment [42]**	Mice: IP 100 μg fusion protein in complete Freund's adjuvant, then	• 88% protection with fusion protein<br>• 13% protection with Fc-FLAG		

Vaccines	Immunization Schedule	Mouse Model	Guinea Pig Model	NHP Model
	boost with 25 µg in incomplete Freund's adjuvant–3 doses	epitope tag		
Ebolavirus Vaccines				
**EBOV**<br>• **live[43-46]**<br>• **irradiated [21, 32, 47]**<br>• **irradiated, in liposomes [21, 47]**<br>• **INA+ UV irradiated, MA [48]**	<u>Mice</u><br>• SC, IM, ID 100 PFU MA-EBOV prior to IP challenge [43-46]<br>• IP 10 µg of irradiated EBOV–3 doses [32]<br>• IM, IV 1.4 µg of irradiated EBOV alone or in liposome–2 doses [47]<br>• IM 5 x 10^4 PFU of INA inactivated MA-EBOV–1 or 2 doses [48]<br><u>NHPs</u>:<br>• IV 194 µg of EBOV encapsulated in liposome–3 doses [21, 47]<br>• SC 50 µg of irradiated EBOV-3 doses[21]	• Complete protection with SC, IM live EBOV; [43-45] protection dependent on CD8+ cells and interferon-α/β receptor and not on B or CD4+ cells [45, 46]<br>• Persistent infection in CD4-depleted or B cell-deficient mice[46]<br>• 25, 45, or 55% protection with IP, IM, or IV irradiated EBOV [32, 47]<br>• Complete protection with IV irradiated liposome-encapsulated EBOV[47]<br>• 77% protection with IM liposome encapsulated EBOV<br>• >80% protection with INA-inactivated EBOV[48]		• No protection with liposome encapsulated EBOV; viremia present [21, 47]<br>• 25% protection with rradiated EBOVin macaques; viremia present in all macaques[21] Neutralizing antibody titers present in 1 surviving macaque immunized with irradiated EBOV
**EBOV ΔVP30 [49]**	<u>Mice</u>: IP 10^6 FFU–2 doses<br><u>Guinea Pigs</u>: IP 10^7 FFU–2 doses	• Complete protection correlated with cellular and humoral responses	Complete protection	

Abbreviations: AD: adenovirus, CAGopt: cytomegalovirus early enhancer element and chicken beta-actin promoter optimized, TAFV: Cote d'Ivoire ebolavirus species, CMV: cytomegalovirus promoter, CTL: Cytotoxic T lymphocytes, Ctr: C terminal truncation, EBOV: Zaire ebolavirus species, F: fusion protein, FFU: focus-forming units, FLAG: DTKDDDDK peptide fused to Fc region of IgG1, GCD: glycoprotein cytoplasmic domain, GM-CSF: granulocyte macrophage colony stimulating factor,GP: glycoprotein, GPA: guinea pig adapted, HIV: human immunodeficiency virus, HN: hemagglutinin-neuraminidase, HPIV3: human parainfluenza virus type 3, ID: intradermal, IFU: infectious units, Ig: Immunoglobulin, IFN-γ: interferon gamma, IM: intramuscular, IN: intranasal, INA: 1,5-iodonaphthylazide, IP: intraperitoneally, IT: intratracheal, IV: Intravenous, KO: knockout, KUN: Kunjin, MA: mouse adapted, mAb: monoclonal antibody, MARV: Marburg virus, NHP: nonhuman primate, NOD: nonobese diabetic, NP: nucleoprotein, PA: protective antigen, PFU: plaque-forming units, PIC: polyinosinic:polycytidylic acid, PO: oral, RABV: rabies virus, rBV: recombinant baclovirus, RESTV: Reston ebolavirus species, RNA: ribonucleic acid, SC: subcutaneous, SCID: severe combined immunodeficiency, SUDV: Sudan ebolavirus species, sGP: soluble glycoprotein, STAT-1: signal transducer and activator of transcription-1 protein, TCID: tissue culture infective dose, TCR: T cell receptor, Th1: T helper cells 1 subset, VEEV: Venezuelan equine encephalitis virus, VLP: virus-like particles, VP: viral protein, VRPs: VEEV RNA replicon particles, VSV: vesicular stomatitis virus, VV: vaccinia virus

Supplemental Table 2. Efficacy of Peri-exposure Treatment in Animal Models of EVD.

Peri-exposure Treatment	Dose and Route of Administration	Mouse Model	Guinea Pig Model	HP Model
**EBOV**<br>• **Live [50]**<br>• **Irradiated [51]**<br>• **INA- inactivated, MA [48]**	Mice<br>• SC 10^1–10^6 PFU EBOV - 18–48 hours prior to IP exposure[50]<br>• IP 5 x 10^4 PFU of INA - inactivated EBOV -3 days [48]<br>• IP 25 µg of irradiated EBOV -3 days	• Protection dependent on SC dose and time to challenge; complete protection at highest dose and greatest time interval to challenge[50]<br>• Complete protection with inactivated EBOV [48]<br>• No protection with irradiated EBOV [51]		
Virus Vectors				
**Ad5 Immunogens [9]**<br>• **CMVEBOV GP**<br>• **CAGopt EBOV GP**	Mice: IM 5 x 10^7 IFU +30 minutes	• Complete protection with AD5/CAGopt EBOV GP<br>• 22% survived with AD/CMVEBOV GP		
**VSV ΔGP Immunogens**<br>• **EBOV GP [52]**<br>• **SUDV GP [53]**	Mice: IP 2 x 10^5 VSV ΔGP/EBOV GP PFU -1 day or +30 minutes or 1 day [52]<br>Guinea pigs: IP 2 x 10^5 VSV ΔGP/EBOV GP PFU -24 hours or +1 or 24 hours [52]<br>NHPs:<br>IM 2 x 10^7 PFU of VSV ΔGP/EBOV GP [52] or VSV ΔGP/SUDV GP +20–30 minutes postexposure to EBOV or SUDV [53]	• Complete protection with VSV ΔGP/EBOV GP regardless of time of treatment [52]<br>• Mild weight loss on +1 day, suggesting viral replication	• 66, 83, or 50% protection with VSV ΔGP/EBOV GP 24 hours prior to or 1 or 24 hours after challenge, respectively [52]	• 50% protection with VSV ΔGP/EBOV GP +20–30 minutes [52]<br>• Complete protection with VSV ΔGP/SUDV GP +20–30 minutes [53]<br>• Control subject receiving VSV ΔGP/LASV GP lived for 17 days after SUDV challenge
Virus-like Particles (VLPs)				

Peri-exposure Treatment	Dose and Route of Administration	Mouse Model	Guinea Pig Model	HP Model
• **EBOV GP + VP40[51]**<br>• **EBOV VP40**	Mice:<br>• IM, IP 25 µg of EBOV GP + VP40 VLPs -1–3 days<br>• 10 µg of EBOV VP40 VLPs -3 days	• 80–100% protection EBOV GP + VP 40<br>• Complete protection with EBOV VP40 VLPs only; not dependent on presence of EBOV GP<br>• 15–17% protection in NK cell-deficient or -depleted mice		
Passive Immunity				
**Pooled immune serum to live EBOV [43, 54]**	Immunocompetent mice: IP 1 mL of antisera (anti-EBOV IgG titers of ≥6,400) _1 day or + 1 day [43]<br>SCID mice: IP 1 mL of antisera (anti-EBOV IgG of ≥400,000 titers) -1 day<br>NHPs: IV 6 mL/kg whole blood immediately after challenge and +3 or 4 days (anti-EBOV IgG ELISA titers of 100,000) [54]	• 89% protection in immunocompetent mice pretreated with immune serum [43]<br>• Complete protection in immunocompetent mice from postchallenge treatment with immune serum<br>• Complete protection in SCID mice pretreated with immune serum<br>• Protection correlated with anti-EBOV IgG titers		• No protection or delay in death compared to controls [54]<br>• Rapid decline of anti-EBOV IgG titers by day +3<br>• Comparable viremia in treated and control NHPs
**Pooled immune serum to VSV ΔGP/EBOV GP [15]**	Mice: IP 0.5 mL of immune serum -1 day	• 80% protection with pooled immune serum; neutralizing antibody titers equivocal		

Peri-exposure Treatment	Dose and Route of Administration	Mouse Model	Guinea Pig Model	HP Model
**Pooled Immune serum to VLPs expressing:**<br>• **EBOV NP [22, 23]**<br>• **EBOV GP [23, 27]**<br>• **EBOV VP24 [27]**<br>• **EBOV VP30**<br>• **EBOV VP40**<br>• **EBOV VP40 + GP[33]**	Mice<br>• IP 0.8 mL of antisera (~4 $\log_{10}$ ELISA titer) to VRP/EBOV GP or NP-9 hours [23]<br>• IP 1mL of antisera to VRP/EBOV NP (2.5-3 $\log_{10}$ ELISA titer), [22] VRP/EBOV GP, VRP/EBOV VP24, VRP/EBOV VP30, or VRP/EBOV VP40 (~4 $\log_{10}$ ELISA titer) [27] -1 day [22, 27]<br>• IV 0.5 mL of antisera to VLP/EBOV VP40 and GP -3 days [33]<br>Guinea pigs: IP 5 mL of antisera (4 $\log_{10}$ ELISA titer) to VRP/EBOV GP -3 hours [23]	• 10% protection with antisera to VRP-EBOV NP [23]<br>• 75–85% protection with antisera to VLP-EBOV GP [27] not confirmed with antisera with same ELISA titers in similar study [23]<br>• No protection with antisera to VLP/EBOV VP40 and GP, [33] VRP/EBOV NP, [22], or VRP-EBOV VP proteins [27]<br>• Lack of protection could be due to a poor CTL response (not measured)	• 20% protection with transfer of immune serum to VRP/EBOVGP [23]<br>• Cell mediated protection may be more important than humoral responses	
**Purified polyclonal IgG antibody against:**<br>• **live EBOV [43]**<br>• **DNA and rAd5 EBOV GP vectors [55]**<br>• **Unknown, EBOV-immunized horses [56, 57]**	Mice:<br>• IP 1 mL of purified mouse IgG (>100,000–400,000 anti-EBOV IgG titers) -1 day [43]<br>• SC 0.03, 0.3, 3 mL/kg horse IgG +20–30 minutes [57]<br>Guinea Pigs: IM 1 mL/kg	• 40-66% protection with mouse IgG[43]<br>• Efficacy of protection with IgG antibodies is titer dependent<br>• Similar protection with polyclonal IgG or immune serum transfer with equivalent IgG titers	• Complete protection with horse IgG given at day 0 only; no viremia detected [57]<br>• Complete protection with horse IgG with second dose at day +3; viremia not detected<br>• No protection if IgG is	• 25% protection with IgG from NHPs immunized with DNA and rAd5 EBOV GP vectors [55]<br>• No protection with horse IgG immediately postchallenge[56, 57] or -2 days [57]<br>• Delayed viremia with

Peri-exposure Treatment	Dose and Route of Administration	Mouse Model	Guinea Pig Model	HP Model
	several minutes and +3 days postexposure or +4 days only[57]<br>NHPs:<br>• IV 160-600 mg NHP IgG/kg -6 or 16 hours [55]<br>• IM ~1 mL/kg of horse IgG (log serum neutralization index of 4.2) immediately after challenge [56, 57], or -2 days prior to or day 0 and day +5 [57]	• 25% protection with horse IgG at highest dose only; lower doses not effective [57]	delayed until day +4; transient reduction in viremia and anti-EBOV titers not detectable	reduction in anti-EBOV titers with NHPs receiving IgG immediately after challenge; no delay in death<br>• 33% protection with 2 doses of horse IgG
**mAb EBOV GP-specific**<br>• **Mouse IgG2a [58, 59]**<br>• **Mouse IgG1 [59, 60]**<br>• **Mouse IgG2b [59]Human IgG1[61, 62]**<br>• **Chimeric human IgG1-mouse IgG2a [63]**	Mice:<br>• IP 25, 50, 100 µg of mAB IgG2a or IgG1 -1 day or +1–2 days [58]<br>• IP 2–256 µg of mouse IgG1 -1 day and +2 days [60]<br>• IP 100 µg of mouse IgGa, IgG2b, or IgG1 ±1 day [59]<br>• IP 100 µg of mouse IgG1 -1 day or +2–4 days [60]<br>• IP 3 µg or greater of chimeric glycoforms -1 day [63]<br>Guinea Pigs:<br>• IP 0.5, 5, 50 mg/kg of human IgG1	• Dose-dependent protection with mouse IgG2a or IgG1 [58, 60]<br>• Mouse IgG2a more effective (20-100%) than IgG1 (0-60%) given ±1 day [58]; not confirmed in recent study [59]<br>• Protection not correlated with neutralizing capacity [58, 59]<br>• Mouse IgG2a, IgG2b, or IgG1 more effective given after challenge than before challenge [59]<br>• 75–88% protection with 1 dose of mouse IgG1 given prior to or +2 days [60]	• No protection when Human mAb given +6 hours [61]<br>• 100% protection at highest dose (50 mg/kg) when human mAb given at time of challenge or -1 hour (25 mg/kg)<br>• 80% protection if human mAb given +1 hour<br>• 25–66% protection with mouse IgG1 133/3.16 given -1 day or +2 days after challenge; little protection with mAb 266/8.1 [60]	• No protection with human mAb [62]<br>• Minimal effect on EBOV viral replication<br>• Cellular immunity may be needed for protection

Peri-exposure Treatment	Dose and Route of Administration	Mouse Model	Guinea Pig Model	HP Model
	(neutralizing) several minutes postchallenge [61]<br>• IP 25 mg/kg of human IgG1 -1 hour or +1 or 6 hours<br>• IP 20–25 mg/kg of mouse IgG1 given -1 day or up to +2 days [60]<br>• IP 10–12.5 mg/kg of mouse IgG1 on days +1, 3, 5, 7, and 9<br>NHPs: IV 50 mg/kg -1 day and +4 days [62]	• Dose-dependent protection with chimeric mouse human mAb fucose-free glycoform [63]	• Multiple lower doses of mouse IgG1 postexposure not improve protection compared to higher dose close to challenge	
**Murine immune components to VLPs/EBOV VP40 and GP:**<br>• **Immune serum**<br>• **Splenocytes**<br>• **T or B cells [33]**	Mice: IV 2 x 10^7 unfractionated splenocytes and/or 0.5 mL of immune serum or1 x 10^7 B or T cells -3 days	• No protection with splenocytes, immune serum, or T or B cells from immunized mice<br>• 90% protection with transfer of both immune serum and splenocytes		
**CD8+ T cells from mice vaccinated withVRP/EBOV GP, NP, or VP proteins and restimulated with peptides fromthese proteins [24]**<br>**T cells (CD4+-or CD8+-enriched, or unfractionated T cells from VRP EBOV NP**	Mice: IP 1 x 10^4–8 x 10^6 T cells -4 hours [22, 24]	• Complete protection with unfractionated T cells [22]<br>• Protection with CD8+ cells dependent on epitopes present in EBOV proteins and MHC class I molecules expressed by different mouse strains[24]<br>• No protection with CD4+-enriched cells [22]		

Peri-exposure Treatment	Dose and Route of Administration	Mouse Model	Guinea Pig Model	HP Model
**vaccinated mice[22]**				
**NK cells stimulated with EBOV GP + VP40 or EBOV VP40 VLPs [51]**	Mice: IP adoptive transfer of 5 x 10^6 stimulated NK cells -6 hours	• 70% protection with transfer of NK cells stimulated with EBOV GP + VP40 VLPs; dependent on perforin, not dependent on IFN-γ<br>• 100% protection with transfer of NK cells stimulated with VLPs/EBOV VP40		
Antiviral Agents				
**FGI-103 [64]**	Mice:<br>• IP 10 mg/kg -1 hour and days +2 and 5<br>• IP 5 or 10 mg/kg +1 day<br>• IP 10 mg/kg once +1–5 days	• Complete protection with 3 doses<br>• Dose-dependent protection (60-100%) with single dose +1 day<br>• No protection with single dose +2 or more days		
**FGI-106 [65]**	Mice:<br>• IP 01, 0,5, 1, 2, 5 mg/kg of FGI-106 -1 hour and +24 and 72 hours postchallenge[65]<br>• IP 5 mg/kg given on day +1; days +1 and 5; or days 0, +1, 3, and 5<br>• IP 0.5, 1, 5 mg/kg single dose on day +1	• Dose-dependent protection when given before and after challenge; complete protection with 2 higher dosages<br>• Complete protection with 5 mg/kg given on day +1 and 5, and day 0, +1, 3, and 5<br>• Dose dependent protection with single doses given +1 day; 90% protection with single 1 or 5 mg/kg dose		

Peri-exposure Treatment	Dose and Route of Administration	Mouse Model	Guinea Pig Model	HP Model
		• Dramatic reduction in tissue viral load		
**Cyanovirin-N [66]**	Mice: SC 0.48, 1.4, 4.8, or5.6 mg/kg -1 day before or at challenge, then daily for +5-8 days	• 20–40% protection; mean time to death increased at 2 higher dosages		
**3-DeazaneplanocinA [45, 67]**	Immunocompetent Mice:<br>• SC 1 mg/kg +1 hour or +1 or 2 days [45, 67] or +3 or 4 days [67]<br>• SC 1 mg/kg day +1, days +1–2, or days +1–3 [67]<br>• SC 0.125, 0.25, 0.5, or 1 mg/kg given once +1 day [67]<br>SCID mice: SC 1 mg/kg single dose day 0–3 postchallenge or days +1–15 [67]	• Dose-dependent protection (40-100%) in immunocompetent mice treated with single dose[67]<br>• Complete protection in immunocompetent mice if treated once within 2 days of challenge; [45, 67] dependent on IFN-α/β [45]<br>• Delay of death in SCID mice with single or multiple doses postinoculation [67]		
**Carbocyclic 3-deazaadenosine[67, 68]**	Immunocompetent Mice:<br>• SC 10, 20, 40, 80 mg/kg once on day +1 [67]<br>• SC 80 mg/kg single dose on day 0–4 [67]<br>• IP 0.03–20 mg/kg initiated -1 day continuing every 8 hours for 9 days [68]<br>• IP 2.2, 6.7, or 20 mg/kg initiated 0–3 days continuing every 8 hours for 5–9 days	• Dose-dependent protection (0-100%) in immunocompetent mice given a single dose[67, 68]<br>• Nearly complete protection with single SC dose (80 mg/kg) given on days +1–2 [67]<br>• Reduction in viral titer greatest with single dose given on day +2<br>• Delay of death in SCID mice<br>• Complete protection with		

Peri-exposure Treatment	Dose and Route of Administration	Mouse Model	Guinea Pig Model	HP Model
	SCID mice: SC 80 mg/kg single dose on day 0–3 [67]	IP dosages ≥0.7 mg/kg initiated -1 day [68] Complete protection with 2 lower IP dosages given at day 0 or +1 day		
**rhuman mannose-binding lectin (rhMBL) [69]**	Mice: IP 75 or 350 µg of rhMBL every 12 hours for 10 days initiated _1 hour or +12 hours	• No protection with lower dosage<br>• >40% protection with pre- or post-treatment<br>• No protection in C3 KO mice		
**Antisense Phosphorodiamidate morpholino oligomers (PMOs)[70-73]**	Mice:<br>• IP 5, [72] 50,[72] or 500 [70, 72] µg of PMO targeting VP35 at -24 and 4 hours<br>• IP 1, 5, or 50 [71, 72] or 500 ug[72]of PMOs targeting VP24 at -24 and 4 hours [71, 72]<br>• IP 5, 50, or 500 µg of 3 PMOs targeting VP24, VP35 or L -4 hours or +24 hours [72]<br>• IP 50 or 500 µg of PMO targeting L L -4 hours or -24 hours<br>• IP 10 mg/kg of PMO with piperazine moieties targeting VP24 and VP35 -24 hours or +24, 48, 72 or 96 hours [73]	• Complete protection following pretreatment with 500 µg (2 doses) of PMO targeting VP35 [70, 72]<br>• 100% protection following pretreatment with 2 higher doses PMOs targeting VP24, depending on location of homologous sequences [71]<br>• Nearly complete protection following pretreatment with 500 ug of PMO targeting VP24[72]<br>• Complete protection with highest dose of 3 PMOs each targeting VP24,VP35, or L either pre- or postexposure[72]<br>• ~30% protection with	• <75% protection with combination of PMOs each targeting VP24, VP35, or L given +4 days; protection lower with given preexposure or +1 day [72]<br>• Reduction in viral titer correlated with survival	• 50% protection with PMOs targeting VP24+VP35+L [72]<br>• High anti-EBOV antibodies and T cell responses in survivors<br>• No protection with PMO targeting VP35 only<br>• 62.5% protection with SC and IP piperazine-enriched PMOs targeting VP24 and VP35 [73]<br>• Reduced viremia and release of IL-6 and MCP-1 with PMOs targeting VP24 and VP35<br>• Dose dependent protection (0-60%) with IV PMOs targeting VP24 and VP35<br>• 100 times lower viral

Peri-exposure Treatment	Dose and Route of Administration	Mouse Model	Guinea Pig Model	HP Model
**Antisense PMOs (continued)**	Guinea Pigs: IP 10 mg of each PMO targeting VP24, VP35 or L -1 day or +1 or 4 days [72]<br>NHPs:<br>• SC, IP, and IM of PMO(s) targeting VP35 or VP24+VP35+L -2 days through +9 days [72]<br>• SC and IP of piperazine-enriched PMOs 40 mg/kg targeting VP24 and VP35 30–60 minutes after challenge then daily for +10 or 14 days[73]<br>• IV 4, 16, 28, or 40 mg/kg of PMOs targeting VP24 and VP35 30–60 minutes after challenge then daily for +14 days	following pretreatment with 500 ug of PMO targeting L<br>• Complete protection following pretreatment with PMO targeting VP24 and VP35 [72, 73]<br>• Postexposure protection diminishes with delay of administration of piperazine-enriched PMOs targeting VP24 and VP35[73]		titers in treated NHPs than in NHPs receiving PMO targeted to MARV proteins
**Small interfering RNA (siRNA) encapsulated in liposomes [74, 75]**	Guinea Pigs:<br>• IP 8 mg/kg of a pool of 4 siRNAs targeting L in polyethylenimine -3 hours prior to challenge then +1, 2, and 4 days [74]<br>• IP 0.75 or 1 mg/kg of pool of 4 siRNAs targeting L in SNALP +1 hour then daily for +6 days		• 20% protection with polyethylenimine-delivered siRNAs targeting L gene; reduction in viremia following siRNA administration [74]<br>• 60% protection with higher dosage of SNALP-delivered siRNAs targeting L gene<br>• Complete protection	• 66% protection with 4 doses of siRNAs targeting EBOV L, VP 24, and VP 35; induced mRNA cleavage at target sites; no viremia in survivors[75]<br>• Complete protection with 7 doses of siRNAs; low EBOV viremia in treated animals

Peri-exposure Treatment	Dose and Route of Administration	Mouse Model	Guinea Pig Model	HP Model
**siRNA encapsulated in liposomes (continued)**	<u>NHPs</u>:<br>• IV 2 mg/kg of pool of siRNAs targeting EBOV L, VP 24, and VP 35 in SNALP +30 minutes, day +1, 3, and 5 after challenge–4 doses total [75]<br>• IV 2 mg/kg of pool of siRNAs in SNALP +30 minutes and daily for days +1–6 –7 doses total		with lower dosage of SNALP-delivered siRNAs pool; no viremia detected	

Abbreviations: AD: adenovirus, CAGopt: cytomegalovirus early enhancer element and chicken beta-actin promoter optimized, CMV: cytomegalovirus promoter, CTL: Cytotoxic T lymphocytes, EBOV: Zaire ebolavirus species, ELISA: enzyme-linked immunosorbent assay, FGI-103: (2-(2-(5-amino(imino)methyl)-1-benzofuran-2-yl)vinyl)-1H-benzoimidazole-5-carboximidamide), FGI-106: (quino [8,7-h] quinoline-1,7diamine,N,N[illegible]-bis [3-(dimethylamino)propyl]-3,9-dimethyl-, tetrahydrochloride), GP: glycoprotein, Ig: Immunoglobulin,IL-6: interleukin-6, IFN: interferon, IM: intramuscular, INA: 1,5-iodonaphthylazide, IP: intraperitoneally, IV: intravenous, KO: knockout, mAb: monoclonal antibody, L: L polymerase, LASV: Lassa virus, MCP-1: monocyte chemotactic protein-1, MHC: major histocompatibility complex, NHP: nonhuman primate, NK: natural killer cells, NP: nucleoprotein, PFU: plaque-forming units, PMO: antisense phosphorodiamidate morpholino oligomers, rhMBL: recombinant human mannose-binding lectin, RNA: ribonucleic acid, SC: subcutaneous, SCID: severe combined immunodeficiency, SUDV: Sudan ebolavirus species, siRNA: small interfering RNAs, SNALP: stable nucleic acid lipid particle, VLP: virus-like particles, VP: viral protein, VRPs: VEEV RNA replicon particles, VSV: vesicular stomatitis virus

References

1. Bukreyev, A.; Marzi, A.; Feldmann, F.; Zhang, L.; Yang, L.; Ward, J.M.; Dorward, D.W.; Pickles, R.J.; Murphy, B.R.; Feldmann, H.; *et al*. Chimeric human parainfluenza virus bearing the Ebola virus glycoprotein as the sole surface protein is immunogenic and highly protective against Ebola virus challenge. *Virology* **2009**, *383*, 2, 348–361.
2. Bukreyev, A.; Yang, L.; Zaki, S.R.; Shieh, W.J.; Rollin, P.E.; Murphy, B.R.; Collins, P.L.; Sanchez, A. A single intranasal inoculation with a paramyxovirus-vectored vaccine protects guinea pigs against a lethal-dose Ebola virus challenge. *J. Virol.* **2006**, *80*, 2267–2279.
3. Bukreyev, A.; Rollin, P.E.; Tate, M.K.; Yang, L.; Zaki, S.R.; Shieh, W.J.; Murphy, B.R.; Collins, P.L.; Sanchez, A. Successful topical respiratory tract immunization of primates against Ebola virus. *J. Virol.* **2007**, *81*, 6379–6388.
4. Blaney, J.E.; Wirblich, C.; Papaneri, A.B.; Johnson, R.F.; Myers, C.J.; Juelich, T.L.; Holbrook, M.R.; Freiberg, A.N.; Bernbaum, J.G.; Jahrling, P.B.; *et al*. Inactivated or live-attenuated bivalent vaccines that confer protection against rabies and Ebola viruses. *J. Virol.* **2011,** *85*, 10605–10616.
5. Croyle, M.A.; Patel, A.; Tran, K.N.; Gray, M.; Zhang, Y.; Strong, J.E.; Feldmann, H.; Kobinger, G.P. Nasal delivery of an adenovirus-based vaccine bypasses pre-existing immunity to the vaccine carrier and improves the immune response in mice. *PloS one* **2008,** *3*, e3548.
6. Patel, A.; Zhang, Y.; Croyle, M.; Tran, K.; Gray, M.; Strong, J.; Feldmann, H.; Wilson, J.M.; Kobinger, G.P. Mucosal delivery of adenovirus-based vaccine protects against Ebola virus infection in mice. *J. Infect. Dis* **2007,** *196*, S413–S420.
7. Wang, D.; Raja, N.U.; Trubey, C.M.; Juompan, L.Y.; Luo, M.; Woraratanadharm, J.; Deitz, S.B.; Yu, H.; Swain, B.M.; Moore, K.M.; *et al*. Development of a cAdVax-based bivalent ebola virus vaccine that induces immune responses against both the Sudan and Zaire species of Ebola virus. *J. Virol.* **2006**, *80*, 2738–2746.
8. Richardson, J.S.; Abou, M.C.; Tran, K.N.; Kumar, A.; Sahai, B.M.; Kobinger, G.P. Impact of systemic or mucosal immunity to adenovirus on Ad-based Ebola virus vaccine efficacy in guinea pigs. *J. Infect. Dis* **2011,** *204*, S1032–S1042.
9. Richardson, J.S.; Yao, M.K.; Tran, K.N.; Croyle, M.A.; Strong, J.E.; Feldmann, H.; Kobinger, G.P. Enhanced protection against Ebola virus mediated by an improved adenovirus-based vaccine. *PloS one* **2009,** *4*, e5308.
10. Kobinger, G.P.; Feldmann, H.; Zhi, Y.; Schumer, G.; Gao, G.; Feldmann, F.; Jones, S.; Wilson, J.M. Chimpanzee adenovirus vaccine protects against Zaire Ebola virus. *Virology* **2006**, *346*, 394–401.
11. Roy, S.; Zhi, Y.; Kobinger, G.P.; Figueredo, J.; Calcedo, R.; Miller, J.R.; Feldmann, H.; Wilson, J.M. Generation of an adenoviral vaccine vector based on simian adenovirus 21. *J. Gen. Virol.* **2006**, *87*, 2477–2485.
12. Marzi, A.; Ebihara, H.; Callison, J.; Groseth, A.; Williams, K.J.; Geisbert, T.W.; Feldmann, H. Vesicular stomatitis virus-based Ebola vaccines with improved cross-protective efficacy. *J. Infect. Dis* **2011,** *204*, S1066–S1074.

13. Garbutt, M.; Liebscher, R.; Wahl-Jensen, V.; Jones, S.; Moller, P.; Wagner, R.; Volchkov, V.; Klenk, H.D.; Feldmann, H.; Stroher, U. Properties of replication-competent vesicular stomatitis virus vectors expressing glycoproteins of filoviruses and arenaviruses. *J. Virol.* **2004**, *78*, 5458–5465.
14. Geisbert, T.W.; Daddario-Dicaprio, K.M.; Lewis, M.G.; Geisbert, J.B.; Grolla, A.; Leung, A.; Paragas, J.; Matthias, L.; Smith, M.A.; Jones, S.M.; *et al.* Vesicular stomatitis virus-based ebola vaccine is well-tolerated and protects immunocompromised nonhuman primates. *PLoS Pathogens* **2008,** *4*, e1000225.
15. Jones, S.M.; Stroher, U.; Fernando, L.; Qiu, X.; Alimonti, J.; Melito, P.; Bray, M.; Klenk, H.D.; Feldmann, H. Assessment of a vesicular stomatitis virus-based vaccine by use of the mouse model of Ebola virus hemorrhagic fever. *J. Infect. Dis* **2007,** *196*, S404–S412.
16. Qiu, X.; Fernando, L.; Alimonti, J.B.; Melito, P.L.; Feldmann, F.; Dick, D.; Stroher, U.; Feldmann, H.; Jones, S.M. Mucosal immunization of cynomolgus macaques with the VSVDeltaG/ZEBOVGP vaccine stimulates strong ebola GP-specific immune responses. *PloS One* **2009,** *4*, e5547.
17. Geisbert, T.W.; Daddario-Dicaprio, K.M.; Geisbert, J.B.; Reed, D.S.; Feldmann, F.; Grolla, A.; Stroher, U.; Fritz, E.A.; Hensley, L.E.; Jones, S.M.; *et al*. Vesicular stomatitis virus-based vaccines protect nonhuman primates against aerosol challenge with Ebola and Marburg viruses. *Vaccine* **2008**, *26*, 6894–6900.
18. Jones, S.M.; Feldmann, H.; Stroher, U.; Geisbert, J.B.; Fernando, L.; Grolla, A.; Klenk, H.D.; Sullivan, N.J.; Volchkov, V.E.; Fritz, E.A.; *et al*. Live attenuated recombinant vaccine protects nonhuman primates against Ebola and Marburg viruses. *Nat. Med.* **2005**, *11*, 786–790.
19. Tsuda, Y.; Caposio, P.; Parkins, C.J.; Botto, S.; Messaoudi, I.; Cicin-Sain, L.; Feldmann, H.; Jarvis, M.A. A replicating cytomegalovirus-based vaccine encoding a single Ebola virus nucleoprotein CTL epitope confers protection against Ebola virus. *PLoS Neglected Tropical Diseases* **2011,** *5*, e1275.
20. Gilligan, K.; Geisbert, J.; Jahrling, P.; Anderson, K. Assessment of Protective Immunity Conferred by Recombinant Vaccinia Viruses to Guinea Pigs Challenged with Ebola Virus. In *Vaccines 97: Molecular Approaches to the Control of Infectious Diseases*; Brown, F.; Burton, D., Doherty, P., Mekalanos, J., Norrby, E., Eds.; Cold Spring Harbor Laboratory Press: Plainview, NY, USA, 1997; pp 87–92.
21. Geisbert, T.W.; Pushko, P.; Anderson, K.; Smith, J.; Davis, K.J.; Jahrling, P.B. Evaluation in nonhuman primates of vaccines against Ebola virus. *Emerg Infect. Dis* **2002**, *8*, 503–507.
22. Wilson, J.A.; Hart, M.K. Protection from Ebola virus mediated by cytotoxic T lymphocytes specific for the viral nucleoprotein. *J. Virol.* **2001**, *75*, 2660–2664.
23. Pushko, P.; Bray, M.; Ludwig, G.V.; Parker, M.; Schmaljohn, A.; Sanchez, A.; Jahrling, P.B.; Smith, J.F. Recombinant RNA replicons derived from attenuated Venezuelan equine encephalitis virus protect guinea pigs and mice from Ebola hemorrhagic fever virus. *Vaccine* **2000**, *19*, 142–153.
24. Olinger, G.G.; Bailey, M.A.; Dye, J.M.; Bakken, R.; Kuehne, A.; Kondig, J.; Wilson, J.; Hogan, R.J.; Hart, M.K. Protective cytotoxic T-cell responses induced by venezuelan equine encephalitis virus replicons expressing Ebola virus proteins. *J. Virol.* **2005**, *79*, 14189–14196.

25. Phoolcharoen, W.; Dye, J.M.; Kilbourne, J.; Piensook, K.; Pratt, W.D.; Arntzen, C.J.; Chen, Q.; Mason, H.S.; Herbst-Kralovetz, M.M. A nonreplicating subunit vaccine protects mice against lethal Ebola virus challenge. *Proceedings of the National Academy of Sciences of the United States of America* **2011**, *108*, 20695–20700.
26. Pushko, P.; Geisbert, J.; Parker, M.; Jahrling, P.; Smith, J. Individual and bivalent vaccines based on alphavirus replicons protect guinea pigs against infection with Lassa and Ebola viruses. *J. Virol.* **2001**, *75*, 11677–11685.
27. Wilson, J.A.; Bray, M.; Bakken, R.; Hart, M.K. Vaccine potential of Ebola virus VP24, VP30, VP35, and VP40 proteins. *Virology* **2001**, *286*, 384–390.
28. Reynard, O.; Mokhonov, V.; Mokhonova, E.; Leung, J.; Page, A.; Mateo, M.; Pyankova, O.; Georges-Courbot, M.C.; Raoul, H.; Khromykh, A.A.; Volchkov, V.E. Kunjin virus replicon-based vaccines expressing Ebola virus glycoprotein GP protect the guinea pig against lethal Ebola virus infection. *J. Infect. Dis* **2011,** 204, S1060–S1065.
29. Sun, Y.; Carrion, R., Jr.; Ye, L.; Wen, Z.; Ro, Y.T.; Brasky, K.; Ticer, A.E.; Schwegler, E.E.; Patterson, J.L.; Compans, R.W.; Yang, C. Protection against lethal challenge by Ebola virus-like particles produced in insect cells. *Virology* **2009**, *383*, 12–21.
30. Warfield, K.L.; Posten, N.A.; Swenson, D.L.; Olinger, G.G.; Esposito, D.; Gillette, W.K.; Hopkins, R.F.; Costantino, J.; Panchal, R.G.; Hartley, J.L.; *et al*. Filovirus-like particles produced in insect cells: immunogenicity and protection in rodents. *J. Infect. Dis* **2007,** *196*, S421–S429.
31. Swenson, D.L.; Warfield, K.L.; Negley, D.L.; Schmaljohn, A.; Aman, M.J.; Bavari, S. Virus-like particles exhibit potential as a pan-filovirus vaccine for both Ebola and Marburg viral infections. *Vaccine* **2005**, *23*, 3033–3042.
32. Warfield, K.L.; Bosio, C.M.; Welcher, B.C.; Deal, E.M.; Mohamadzadeh, M.; Schmaljohn, A.; Aman, M.J.; Bavari, S. Ebola virus-like particles protect from lethal Ebola virus infection. *Proceedings of the National Academy of Sciences of the United States of America* **2003,** *100*, 15889–15894.
33. Warfield, K.L.; Olinger, G.; Deal, E.M.; Swenson, D.L.; Bailey, M.; Negley, D.L.; Hart, M.K.; Bavari, S. Induction of humoral and CD8+ T cell responses are required for protection against lethal Ebola virus infection. *J. Immunol.* **2005**, *175*, 1184–1191.
34. Raymond, J.; Bradfute, S.; Bray, M. Filovirus infection of STAT-1 knockout mice. *J. Infect. Dis* **2011,** *204,* S986–S990.
35. Warfield, K.L.; Swenson, D.L.; Olinger, G.G.; Kalina, W.V.; Aman, M.J.; Bavari, S. Ebola virus-like particle-based vaccine protects nonhuman primates against lethal Ebola virus challenge. *J. Infect. Dis.* **2007,** *196*, S430–S437.
36. Vanderzanden, L.; Bray, M.; Fuller, D.; Roberts, T.; Custer, D.; Spik, K.; Jahrling, P.; Huggins, J.; Schmaljohn, A.; Schmaljohn, C. DNA vaccines expressing either the GP or NP genes of Ebola virus protect mice from lethal challenge. *Virology* **1998**, *246*, 134–144.
37. Dowling, W.; Thompson, E.; Badger, C.; Mellquist, J.L.; Garrison, A.R.; Smith, J.M.; Paragas, J.; Hogan, R.J.; Schmaljohn, C. Influences of glycosylation on antigenicity, immunogenicity, and protective efficacy of ebola virus GP DNA vaccines. *J. Virol.* **2007**, *81*, 1821–1837.

38. Sullivan, N.J.; Sanchez, A.; Rollin, P.E.; Yang, Z.Y.; Nabel, G.J. Development of a preventive vaccine for Ebola virus infection in primates. *Nature* **2000**, *408*, 605–609.
39. Xu, L.; Sanchez, A.; Yang, Z.; Zaki, S.R.; Nabel, E.G.; Nichol, S.T.; Nabel, G.J. Immunization for Ebola virus infection. *Nat. Med.* **1998**, *4*, 37–42.
40. Riemenschneider, J.; Garrison, A.; Geisbert, J.; Jahrling, P.; Hevey, M.; Negley, D.; Schmaljohn, A.; Lee, J.; Hart, M.K.; Vanderzanden, L.; *et al.* Comparison of individual and combination DNA vaccines for B. anthracis, Ebola virus, Marburg virus and Venezuelan equine encephalitis virus. *Vaccine* **2003,** 21, (25–26), 4071–80.
41. Mellquist-Riemenschneider, J.L.; Garrison, A.R.; Geisbert, J.B.; Saikh, K.U.; Heidebrink, K.D.; Jahrling, P.B.; Ulrich, R.G.; Schmaljohn, C.S. Comparison of the protective efficacy of DNA and baculovirus-derived protein vaccines for EBOLA virus in guinea pigs. *Virus Res.* **2003**, *92*, 187–193.
42. Konduru, K.; Bradfute, S.B.; Jacques, J.; Manangeeswaran, M.; Nakamura, S.; Morshed, S.; Wood, S.C.; Bavari, S.; Kaplan, G.G. Ebola virus glycoprotein Fc fusion protein confers protection against lethal challenge in vaccinated mice. *Vaccine* **2011**, *29*, 2968–2677.
43. Gupta, M.; Mahanty, S.; Bray, M.; Ahmed, R.; Rollin, P.E. Passive transfer of antibodies protects immunocompetent and imunodeficient mice against lethal Ebola virus infection without complete inhibition of viral replication. *J. Virol.* **2001**, *75*, 4649–4654.
44. Bray, M.; Davis, K.; Geisbert, T.; Schmaljohn, C.; Huggins, J. A mouse model for evaluation of prophylaxis and therapy of Ebola hemorrhagic fever. *J. Infect. Dis* **1998**, *178*, 651–661.
45. Bray, M. The role of the Type I interferon response in the resistance of mice to filovirus infection. *J. Gen. Virol.* **2001**, *82*, 1365–1373.
46. Gupta, M.; Mahanty, S.; Greer, P.; Towner, J.S.; Shieh, W.J.; Zaki, S.R.; Ahmed, R.; Rollin, P.E. Persistent infection with ebola virus under conditions of partial immunity. *J. Virol.* **2004,** *78*, 958–967.
47. Rao, M.; Bray, M.; Alving, C.R.; Jahrling, P.; Matyas, G.R. Induction of immune responses in mice and monkeys to Ebola virus after immunization with liposome-encapsulated irradiated Ebola virus: Protection in mice requires CD4(+) T cells. *J. Virol.* **2002**, *76*, 9176–9185.
48. Warfield, K.L.; Swenson, D.L.; Olinger, G.G.; Kalina, W.V.; Viard, M.; Aitichou, M.; Chi, X.; Ibrahim, S.; Blumenthal, R.; Raviv, Y.; *et al*. Ebola virus inactivation with preservation of antigenic and structural integrity by a photoinducible alkylating agent. *J. Infect. Dis* **2007,** *196*, S276–S283.
49. Halfmann, P.; Ebihara, H.; Marzi, A.; Hatta, Y.; Watanabe, S.; Suresh, M.; Neumann, G.; Feldmann, H.; Kawaoka, Y. Replication-deficient ebolavirus as a vaccine candidate. *J. Virol.* **2009**, *83*, 3810–3815.
50. Mahanty, S.; Gupta, M.; Paragas, J.; Bray, M.; Ahmed, R.; Rollin, P.E. Protection from lethal infection is determined by innate immune responses in a mouse model of Ebola virus infection. *Virology* **2003**, *312*, 415–424.
51. Warfield, K.L.; Perkins, J.G.; Swenson, D.L.; Deal, E.M.; Bosio, C.M.; Aman, M.J.; Yokoyama, W.M.; Young, H.A.; Bavari, S. Role of natural killer cells in innate protection against lethal ebola virus infection. *J. Exp. Med.* **2004**, *200*, 169–179.

52. Feldmann, H.; Jones, S.M.; Daddario-DiCaprio, K.M.; Geisbert, J.B.; Stroher, U.; Grolla, A.; Bray, M.; Fritz, E.A.; Fernando, L.; Feldmann, F.; Hensley, L.E.; Geisbert, T.W. Effective post-exposure treatment of Ebola infection. *PLoS Pathogens* **2007,** *3*, e2.
53. Geisbert, T.W.; Daddario-DiCaprio, K.M.; Williams, K.J.; Geisbert, J.B.; Leung, A.; Feldmann, F.; Hensley, L.E.; Feldmann, H.; Jones, S.M. Recombinant vesicular stomatitis virus vector mediates postexposure protection against Sudan Ebola hemorrhagic fever in nonhuman primates. *J. Virol.* **2008**, *82*, 5664–5668.
54. Jahrling, P.B.; Geisbert, J.B.; Swearengen, J.R.; Larsen, T.; Geisbert, T.W. Ebola hemorrhagic fever: Evaluation of passive immunotherapy in nonhuman primates. *J. Infect. Dis* **2007,** *196*, S400–S403.
55. Sullivan, N.J.; Hensley, L.; Asiedu, C.; Geisbert, T.W.; Stanley, D.; Johnson, J.; Honko, A.; Olinger, G.; Bailey, M.; Geisbert, J.B.; *et al*. CD8+ cellular immunity mediates rAd5 vaccine protection against Ebola virus infection of nonhuman primates. *Nat. Med.* **2011**, *17*, 1128–1131.
56. Jahrling, P.B.; Geisbert, J.; Swearengen, J.R.; Jaax, G.P.; Lewis, T.; Huggins, J.W.; Schmidt, J.J.; LeDuc, J.W.; Peters, C.J. Passive immunization of Ebola virus-infected cynomolgus monkeys with immunoglobulin from hyperimmune horses. *Arch. Virol.* **1996**, *11*, 135–140.
57. Jahrling, P.B.; Geisbert, T.W.; Geisbert, J.B.; Swearengen, J.R.; Bray, M.; Jaax, N.K.; Huggins, J.W.; LeDuc, J.W.; Peters, C.J. Evaluation of immune globulin and recombinant interferon-alpha2b for treatment of experimental Ebola virus infections. *J. Infect. Dis* **1999,** *179*, S224–S234.
58. Wilson, J.A.; Hevey, M.; Bakken, R.; Guest, S.; Bray, M.; Schmaljohn, A.L.; Hart, M.K. Epitopes involved in antibody-mediated protection from Ebola virus. *Science* **2000**, *287*, 1664–1666.
59. Qiu, X.; Alimonti, J.B.; Melito, P.L.; Fernando, L.; Stroher, U.; Jones, S.M. Characterization of Zaire ebolavirus glycoprotein-specific monoclonal antibodies. *Clinical immunology* **2011,** *141*, 218–227.
60. Takada, A.; Ebihara, H.; Jones, S.; Feldmann, H.; Kawaoka, Y. Protective efficacy of neutralizing antibodies against Ebola virus infection. *Vaccine* **2007**, *25*, 993–999.
61. Parren, P.W.; Geisbert, T.W.; Maruyama, T.; Jahrling, P.B.; Burton, D.R., Pre- and postexposure prophylaxis of Ebola virus infection in an animal model by passive transfer of a neutralizing human antibody. *J. Virol.* **2002**, *76*, 6408–6412.
62. Oswald, W.B.; Geisbert, T.W.; Davis, K.J.; Geisbert, J.B.; Sullivan, N.J.; Jahrling, P.B.; Parren, P.W.; Burton, D.R. Neutralizing antibody fails to impact the course of Ebola virus infection in monkeys. *PLoS Pathogens* **2007,** *3*, e9.
63. Zeitlin, L.; Pettitt, J.; Scully, C.; Bohorova, N.; Kim, D.; Pauly, M.; Hiatt, A.; Ngo, L.; Steinkellner, H.; Whaley, K.J.; Olinger, G.G. Enhanced potency of a fucose-free monoclonal antibody being developed as an Ebola virus immunoprotectant. *Proceedings of the National Academy of Sciences of the United States of America* **2011**, *108*, 20690–20694.
64. Warren, T.K.; Warfield, K.L.; Wells, J.; Enterlein, S.; Smith, M.; Ruthel, G.; Yunus, A.S.; Kinch, M.S.; Goldblatt, M.; Aman, M.J.; Bavari, S. Antiviral activity of a small-molecule inhibitor of filovirus infection. *J. Infect. Dis* **2010**, *54*, 2152–2159.

65. Aman, M.J.; Kinch, M.S.; Warfield, K.; Warren, T.; Yunus, A.; Enterlein, S.; Stavale, E.; Wang, P.; Chang, S.; Tang, Q.; *et al*. Development of a broad-spectrum antiviral with activity against Ebola virus. *Antivir Res.* **2009**, *83*, 245–251.
66. Barrientos, L.G.; O'Keefe, B.R.; Bray, M.; Sanchez, A.; Gronenborn, A.M.; Boyd, M.R. Cyanovirin-N binds to the viral surface glycoprotein, GP1,2 and inhibits infectivity of Ebola virus. *Antivir Res.* **2003**, *58*, 47–56.
67. Bray, M.; Driscoll, J.; Huggins, J.W. Treatment of lethal Ebola virus infection in mice with a single dose of an S-adenosyl-L-homocysteine hydrolase inhibitor. *Antivir Res.* **2000**, *45*, 135–147.
68. Huggins, J.; Zhang, Z.X.; Bray, M., Antiviral drug therapy of filovirus infections: S-adenosylhomocysteine hydrolase inhibitors inhibit Ebola virus in vitro and in a lethal mouse model. *J. Infect. Dis* **1999,** *179*, S240–S247.
69. Michelow, I.C.; Lear, C.; Scully, C.; Prugar, L.I.; Longley, C.B.; Yantosca, L.M.; Ji, X.; Karpel, M.; Brudner, M.; Takahashi, K.; *et al*. High-dose mannose-binding lectin therapy for Ebola virus infection. *J. Infect. Dis* **2011**, *203*, 175–179.
70. Enterlein, S.; Warfield, K.L.; Swenson, D.L.; Stein, D.A.; Smith, J.L.; Gamble, C.S.; Kroeker, A.D.; Iversen, P.L.; Bavari, S.; Muhlberger, E. VP35 knockdown inhibits Ebola virus amplification and protects against lethal infection in mice. *Antimicrob Agents Chemother* **2006**, *50*, 984–993.
71. Swenson, D.L.; Warfield, K.L.; Warren, T.K.; Lovejoy, C.; Hassinger, J.N.; Ruthel, G.; Blouch, R.E.; Moulton, H.M.; Weller, D.D.; Iversen, P.L.; *et al*. Chemical modifications of antisense morpholino oligomers enhance their efficacy against Ebola virus infection. *Antimicrob Agents Chemother* **2009**, *53*, 2089–2099.
72. Warfield, K.L.; Swenson, D.L.; Olinger, G.G.; Nichols, D.K.; Pratt, W.D.; Blouch, R.; Stein, D.A.; Aman, M.J.; Iversen, P.L.; Bavari, S. Gene-specific countermeasures against Ebola virus based on antisense phosphorodiamidate morpholino oligomers. *PLoS Pathogens* **2006,** *2*, e1.
73. Warren, T.K.; Warfield, K.L.; Wells, J.; Swenson, D.L.; Donner, K.S.; Van Tongeren, S.A.; Garza, N.L.; Dong, L.; Mourich, D.V.; Crumley, S.; *et al*. Advanced antisense therapies for postexposure protection against lethal filovirus infections. *Nat. Med.* **2010**, *16*, 991–994.
74. Geisbert, T.W.; Hensley, L.E.; Kagan, E.; Yu, E.Z.; Geisbert, J.B.; Daddario-DiCaprio, K.; Fritz, E.A.; Jahrling, P.B.; McClintock, K.; Phelps, J.R.; *et al*. Postexposure protection of guinea pigs against a lethal ebola virus challenge is conferred by RNA interference. *J. Infect. Dis* **2006**, *193*, 1650–1657.
75. Geisbert, T.W.; Lee, A.C.; Robbins, M.; Geisbert, J.B.; Honko, A.N.; Sood, V.; Johnson, J.C.; de Jong, S.; Tavakoli, I.; Judge, A.; *et al*. Postexposure protection of non-human primates against a lethal Ebola virus challenge with RNA interference: a proof-of-concept study. *Lancet* **2010**, *375*, 1896–1905.

© 2012 by the authors; licensee MDPI, Basel, Switzerland. This article is an open access article distributed under the terms and conditions of the Creative Commons Attribution license (http://creativecommons.org/licenses/by/3.0/).
